# Determining the Location of the α-Synuclein Dimer Interface Using Native Top-Down Fragmentation and Isotope Depletion-Mass Spectrometry

**DOI:** 10.1021/jasms.2c00339

**Published:** 2023-03-28

**Authors:** Kiani Jeacock, Alexandre Chappard, Kelly J. Gallagher, C. Logan Mackay, David P. A. Kilgour, Mathew H. Horrocks, Tilo Kunath, David J. Clarke

**Affiliations:** † The EastCHEM School of Chemistry, University of Edinburgh, Edinburgh EH9 3FJ, U.K.; ‡ Chemistry and Forensics, Nottingham Trent University, Nottingham NG11 8NS, U.K.; § Centre for Regenerative Medicine, Institute for Stem Cell Research, University of Edinburgh, Edinburgh EH16 4UU, U.K.

## Abstract

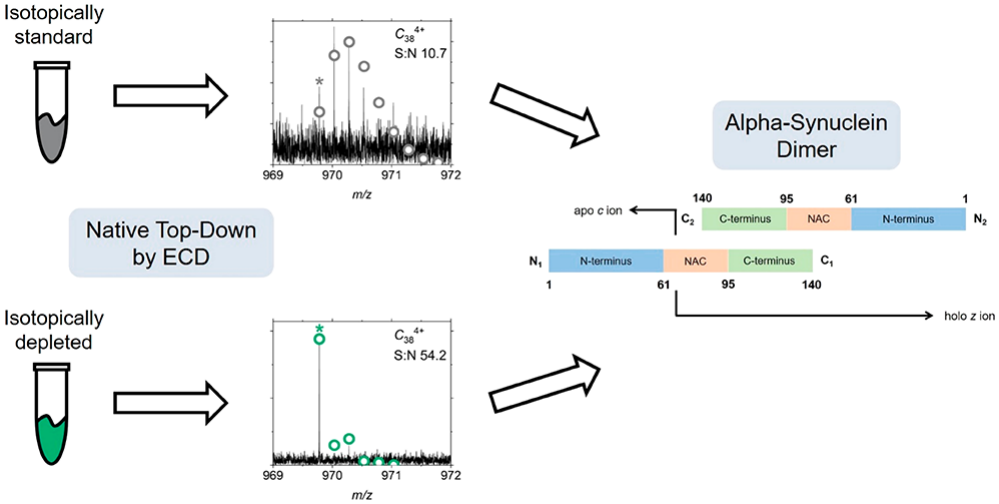

α-Synuclein (αSyn), a 140-residue intrinsically disordered protein, comprises the primary proteinaceous component of pathology-associated Lewy body inclusions in Parkinson’s disease (PD). Due to its association with PD, αSyn is studied extensively; however, the endogenous structure and physiological roles of this protein are yet to be fully understood. Here, ion mobility-mass spectrometry and native top-down electron capture dissociation fragmentation have been used to elucidate the structural properties associated with a stable, naturally occurring dimeric species of αSyn. This stable dimer appears in both wild-type (WT) αSyn and the PD-associated variant A53E. Furthermore, we integrated a novel method for generating isotopically depleted protein into our native top-down workflow. Isotope depletion increases signal-to-noise ratio and reduces the spectral complexity of fragmentation data, enabling the monoisotopic peak of low abundant fragment ions to be observed. This enables the accurate and confident assignment of fragments unique to the αSyn dimer to be assigned and structural information about this species to be inferred. Using this approach, we were able to identify fragments unique to the dimer, which demonstrates a C-terminal to C-terminal interaction between the monomer subunits. The approach in this study holds promise for further investigation into the structural properties of endogenous multimeric species of αSyn.

## Introduction

The small, intrinsically disordered protein α-synuclein (αSyn) plays an important role in the pathogenesis of Parkinson’s disease (PD), forming an integral part of the insoluble inclusions known as Lewy bodies (LBs), found in the midbrain of PD patients.^1^ While the majority of PD cases are idiopathic, around 10% of disease occurrences are linked to genetic mutations, including several single-point mutants which have been identified in the αSyn *SNCA* gene.^2^ These mutations include A30P, E46K, G51D, A53E, and A53T, which are all linked to early onset variations of the disease.^3−7^

Both structural and functional studies of αSyn have primarily focused on the pathogenic role of the protein, as αSyn undergoes a large conformational change from an intrinsically disordered monomer to the ordered amyloid fibrils found in LBs, of which there are multiple high-resolution structures available.^8−12^ This transition is mediated via a series of soluble oligomeric intermediates, which are widely accepted to be the primary cytotoxic constituent.^13−15^ Ion mobility (IM), in combination with mass spectrometry (MS), has proven to be a powerful technique for observing these oligomers *in vacuo*, enabling the determination of protein stoichiometry and collision cross section (CCS), providing insights into the topology and dynamics of these species.^16^ This has been applied successfully to several amyloid-forming proteins including β_2_-microglobulin, amylin, amyloid-β, and αSyn.^17−20^

While IM-MS is an excellent technique for providing global conformational information, the data is too coarse-grained to determine binding interfaces within protein complexes. Electron capture dissociation (ECD) MS, commonly used for proteomics and the localization of post-translational modifications,^21,22^ can be used to derive structural information about proteins, protein complexes, and protein–ligand binding, owing to its ability to retain noncovalent bonds.^23,24^ Therefore, native top-down fragmentation can provide residue-level information regarding the structural properties of higher order oligomers. This was recently shown for the amyloidogenic protein amylin by Lam et al. in 2020, whereby ECD MS was used to determine the location of the interfaces for both the dimer and trimer species of amylin.^25^ Native top-down ECD MS has also been previously applied to αSyn, originally being used to determine interaction sites with spermine, a natural polycation present in neurons.^26^ More recently, ECD MS was performed on both monomeric and dimeric forms of αSyn by Phillips et al. in 2015.^27^ This study demonstrated both a charge-dependent and pH dependent-efficiency of αSyn fragmentation by ECD, with fragmentation being confined almost exclusively to the N-terminus. They postulated this data might represent a dimer interface comprised of the C-terminus; however, the overall number of fragments assigned for this species was minimal, due to their low abundance.^27^

Fragment ions originating from native top-down fragmentation of protein complexes are often of large mass and low abundance, resulting in low signal-to-noise ratios (S/N) and monoisotopic peaks that are not observable. These compounding factors can make confident assignment of these ions challenging. We have recently shown that these difficulties can be mitigated by employing isotope depletion (ID). This strategy was originally demonstrated in 1997,^28^ but an efficient technique for the production of recombinant proteins significantly depleted in ^13^C and ^15^N has been developed in our group more recently.^29^ The use of ID protein samples in top-down fragmentation experiments was shown to increase S/N ratios by reducing spectral complexity and simplifying isotope distributions, thus enabling a large increase in the number of assigned fragment ions for a range of proteins analyzed under denaturing conditions.^29^

Interestingly, higher order species of αSyn are not only associated with disease but may also have a functional, physiological role within the cell. Naturally occurring, endogenous oligomers of αSyn have previously been identified using both antibody-based techniques and MS.^30−32^ A study by Bartels et al. in 2011, provided the first evidence for the existence of a naturally occurring tetrameric αSyn species, which could be isolated from both human cell lines and red blood cell lysates.^30^ This species exhibits an α-helical structure and is resistant to aggregation, suggesting that native αSyn multimers may undergo destabilization prior to misfolding and aggregation into cytotoxic fibrillar structures. They also demonstrated that the lipid-binding ability of the native αSyn tetramer was greatly increased in comparison to recombinantly produced monomer.^30^

Lipid-binding has been suggested as an important functional property of physiological αSyn, and the presence of missense mutations affects the affinity of the protein for lipid membranes.^33^ However, the normal physiological function of αSyn has yet to be fully determined. Putative roles include synaptic vesicle docking,^34^ DNA modulation and damage response,^35,36^ and roles within the neuronal innate immune system.^37,38^ With this in mind, it is of increasing importance to understand the structural and functional properties associated with not only pathogenic oligomers, but also naturally occurring αSyn oligomers, and to determine the effect of mutations on these species.

We hypothesize that by implementing our novel isotope depletion technique into the native top-down ECD MS workflow we will increase the number of assigned fragment ions for endogenous αSyn oligomeric species, such as the dimer. Here, we demonstrate the first use of IM-MS, isotope depletion-mass spectrometry (ID-MS), and native ECD MS to specifically probe the conformational dynamics of naturally occurring multimers derived from wild-type (WT) αSyn and A53E, the most recently identified PD-associated variant.^6^ Using these methods, we can confidently assign enough fragment ions to determine the potential dimer interface of the protein, providing important structural information about this naturally occurring αSyn oligomer.

## Experimental Section

### Molecular Biology

The pT7-7 WT αSyn expression plasmid was kindly donated by the Edinburgh Protein Purification Facility.^39^ The PD-linked single point mutant A53E was prepared via site-directed mutagenesis using the QuikChange Lightning kit (Agilent Technologies). Details of primers used can be found in the Supporting Information, and the mutagenesis was performed following the manufacturer’s protocol. Mutations were confirmed via Sanger sequencing carried out by the MRC Protein Phosphorylation and Ubiquitylation Unit at the University of Dundee.

### αSyn Expression

Isotopically standard proteins were expressed by transforming the required plasmid into *Escherichia coli* BL21 (DE3) cells. A single colony was used to inoculate 10 mL of lysogeny broth (LB) media supplemented with 100 μg/mL ampicillin for overnight incubation at 37 °C with continuous shaking at 180 rpm. Those cells were then used to inoculate 1 L of LB media, which was incubated under the same conditions until an optical density at 600 nm (OD_600_) of 0.6 was reached. Protein expression was then induced using 0.5 mM isopropyl-β-d-1-thiogalactopyranoside (IPTG), and cells were incubated overnight at 18 °C with shaking. After incubation, cells were harvested via centrifugation at 5000 *g* for 30 min at 4 °C. Pellets were stored at −80 °C until required. Isotopically depleted proteins were expressed using 99.5% ^12^C-glucose (Cambridge Isotope Laboratories) and 99.9% ^14^N-ammonium sulfate as the sole carbon and nitrogen sources, as developed and detailed by Gallagher et al.^29^ All other expression conditions were kept the same as for isotopically standard proteins.

### αSyn Purification

Natural abundance isotope αSyn and isotope depleted αSyn were purified using a protocol adapted from Hoyer et al.^40^ Cell pellets were resuspended in 25 mL of Buffer A (20 mM Tris pH 8.0, 1 mM EDTA), subjected to 15 rounds of sonication for 30 s at an amplitude of 10 μm, and then clarified via centrifugation. The clarified lysate was incubated at 85 °C for 10 min and then centrifuged again. Streptomycin sulfate was added to the supernatant to a concentration of 10 mg/mL and incubated at 10 °C for 20 min with agitation. After further centrifugation, ammonium sulfate was added to the supernatant until saturation and then incubated at 10 °C for 20 min with agitation. Following a final centrifugation, the resulting pellet was resuspended in 25 mL of Buffer A, and this was dialyzed against 5 L of the same buffer overnight. The solution was filtered through a 0.22 μm syringe filter then loaded onto a 1 mL HiTrap Q FF column (Cytiva) pre-equilibrated with Buffer A. Unbound protein was removed by washing with Buffer A, and bound proteins were eluted using a linear gradient of 0–100% Buffer B (Buffer A + 1 M NaCl). Fractions containing αSyn were pooled and concentrated using a Vivaspin centrifugal concentrator (MWCO 5 kDa). The concentrated solution was then loaded onto a HiPrep 26/60 Sephacryl S-200 HR column pre-equilibrated with SEC buffer (20 mM Tris pH 7.4, 1 mM EDTA, 100 mM NaCl). Following elution, fractions containing pure αSyn were pooled, and concentration was determined by absorbance at 275 nm using an extinction coefficient of 5600 M^–1^cm^–1^. All proteins were stored in SEC buffer at −80 °C until required. Prior to native mass spectrometry, protein samples were exchanged into 100 mM ammonium acetate using Micro Bio-Spin 6 columns (Bio-Rad).

### Liquid Chromatography–Mass Spectrometry

Intact mass analyses were performed on an Acquity UPLC coupled to a Synapt G2 (Waters). Chromatographic separation was conducted using a 50 × 2.1 mm C4 analytical column (Phenomenex) and an organic gradient of 0–100% acetonitrile and 0.1% (v/v) formic acid was used.

### Ion Mobility-Mass Spectrometry

Ion mobility experiments were conducted on a Synapt G2 (Waters) equipped with a traveling wave ion mobility drift cell, using helium as the drift gas. Experiments were performed in duplicate. Protein samples of 20 μM were desolvated by nanoelectrospray ionization (nESI) using a Triversa Nanomate infusion robot (Advion Biosciences), with typical instrument conditions of capillary voltage 1.3 kV, cone voltage 50 V, trap DC bias voltage 45 V, and source temperature 80 °C. Collision-induced unfolding experiments were performed by recording measurements at incremental trap voltages of 2 V. Experimental collision cross sections (^TW^CCS_He_) were determined by calibrating the drift tube with denatured myoglobin at 0.5 mg/mL, prepared in 50:50 water/acetonitrile and 0.1% formic acid.^41^ Using DriftScope v2.7 (Waters), calibration values with an *R*^2^ > 0.95 were achieved. Data was analyzed using MassLynx v4.1 (Waters) and CIUSuite2.^42^ Gaussian peaks were fitted to the IM-MS data in Origin 2019 using the multiple peak fitting function, with a maximum of 400 iterations and a tolerance of 1e-9.

### Native Top-Down Fragmentation with ECD

For top-down fragmentation with electron capture dissociation, protein samples were prepared in 100 mM ammonium acetate and ionized using a Triversa Nanomate as described above. Spectra were acquired using a SolariX FT-ICR 2XR instrument equipped with a 12 T magnet (Bruker Daltonics). Prior to fragmentation, individual protein charge states were isolated in the mass resolving quadrupole. Ions were accumulated for up to 500 ms in the ICR cell in order to typically achieve a signal of around 10^8^ per scan. ECD cathode conditions were a bias of 1.7 V and a lens voltage of 20 V for monomeric protein and a bias of 1.5 V and a lens voltage of 22 V for the dimeric protein. Typically, an ECD pulse length of between 5 and 15 ms was used.

### Top-Down Data Analysis

Natural isotope abundance FT-ICR data was processed using Data Analysis v4.2 (Bruker Daltonics). The sophisticated numerical annotation procedure (SNAP 2.0) was used for deconvolution, with typical parameters of quality factor threshold of 0.2 and a S/N threshold of 2. The monoisotopic peak list generated by the software was used to search for matching fragments in monomeric data or to search for apo dimer fragments with a mass error of ≤10 ppm in ProSight Lite.^43^ Data generated from isotopically depleted samples was processed in absorption mode using AutoVectis Pro 2023 (Spectroswiss Sarl, Lausanne). Absorption mode spectra were generated using an asymmetric apodization (“Kilgour mode”), with an apex *F* = 0.1.^44,45^ Peak picking was undertaken using a more advanced form of the AutoPiquer algorithm, which is yet to be published but which operates in a manner analogous to that which was previously published.^46^ Assignments were made using the top-down AutoSeequer tool, where the spectra were internally recalibrated using the detected ions, and with a mass error limit of 3 ppm. Assignments were then further revised based on statistical measures of the mass errors within each isotopic envelope, and the relationship between the mass error and the S/N of the peaks. Additionally, assignments which exhibited either anomalously high or low charge states for the size of the fragment were also discounted. Finally, all assignments were manually curated, and with all of the assignments, revision and curation steps were undertaken using the tools built into AutoSeequer. Full details are available on request. Apo dimer fragments were searched in the software by generating a theoretical fragment mass list based on the amino acid sequence of the protein. In order to search for holo dimer fragments (*M* + *c*/*z*), a fixed N- or C-terminal modification was applied to the sequence in order to generate a theoretical mass list. This fixed modification used was the accurate monoisotopic mass of the protein of interest (WT = 14,451.219 Da, A53E = 14,509.225 Da).

## Results and Discussion

### Native Mass Spectrometry Reveals the Presence of a Stable Dimeric Species of αSyn

Wildtype (WT) and A53E αSyn were produced recombinantly in *E. coli* for structural investigation. After expression and purification, both variants were analyzed by native mass spectrometry. For both WT and A53E we observed a wide charge state distribution (CSD), typical for an intrinsically disordered protein (Figure S1). Charge states of the monomeric protein were observed between [M+7H]^7+^ and [M+19H]^19+^ and exhibit a bimodal distribution, centered around both [M+16H]^16+^ and [M+8H]^8+^. A minor species consistent with a dimeric form of αSyn was also observed in both protein variants in charge states ranging from [2M+13H]^13+^ to [2M+21H]^21+^.

**Figure 1:**
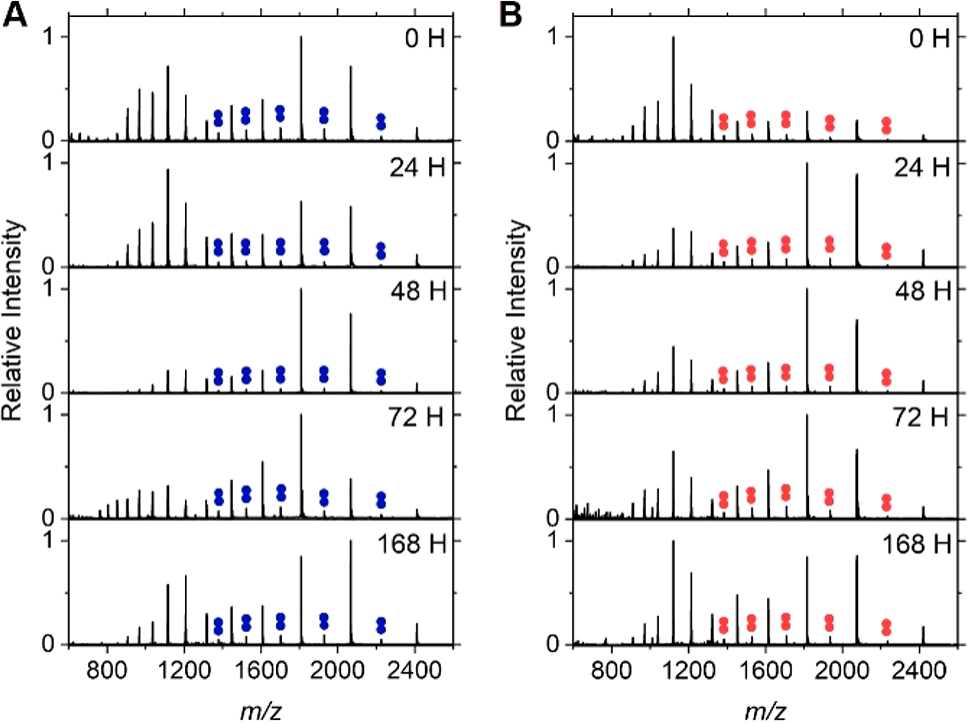
Native charge state distribution (CSD) of αSyn variants over time. Native CSD of (A) WT and (B) A53E αSyn at multiple time points after incubation under aggregating conditions. Conditions were 100 μM protein in 100 mM ammonium acetate at 37 °C with orbital shaking. Samples were not diluted prior to nanoelectrospray ionization to prevent disruption of the monomer:dimer equilibrium. Dimer charge states are indicated in colored double circles, showing a lack of change in intensity over time.

No significant change to the CSD was observed after incubating 100 μM protein, at 37 °C with agitation, conditions known to induce aggregation *in vitro*,^47^ and chosen to be a direct comparison to other aggregation assays. To our surprise, the observed dimer charge states did not change in intensity, and no other higher order species were observed, even up to 168 h of incubation under these aggregation-inducing conditions (Figure 1). For both WT and A53E, we observed a decrease in the intensity of the higher monomeric charge states up to an incubation time of 48 h and then a subsequent increase in intensity again up to the final time point of 168 h. These observations are consistent with changes in the structural properties of monomeric αSyn and changes in the extent of disorder present as the protein is subjected to aggregation-favoring conditions.^48^

Using Thioflavin T, a “gold-standard” fluorophore used to follow the aggregation kinetics of amyloidogenic proteins,^49^ we show that under identical conditions (100 mM ammonium acetate, 100 μM protein) both WT and A53E αSyn aggregate within the 168-h time scale (Figure S2). This suggests that during aggregation higher order species of αSyn are either of too low abundance to be observed by native MS or are unstable and dissociate upon being subjected to nESI. As the charge state intensity, and therefore relative abundance, of the dimer does not change over the time course, we propose that this observed dimeric species might represent a stable naturally occurring oligomer of αSyn, which is incapable of undergoing further oligomerization.

This finding correlates with previous observations of naturally occurring higher order species of αSyn, where minor amounts of dimer, in addition to an α-helical tetramer, were identified.^30^ It should be noted that endogenously expressed αSyn is constitutively N-terminally acetylated, and this may in part explain why no tetrameric species was observed here. In addition, it has also been demonstrated that the missense mutations associated with early onset PD decrease the ability of αSyn to form a helical tetramer and increase the pool of intrinsically disordered monomers (although neither study investigated the A53E variant).^50,51^

### Ion Mobility Mass Spectrometry Highlights Structural Differences between αSyn Variants

To begin, we applied collision-induced dissociation (CID) to the αSyn dimeric species. We performed CID on multiple charge states of the dimer ranging from [2M+13H]^13+^ to [2M+19H]^19+^ and found that even using relatively low voltages of CID, the dimer species immediately dissociated into two corresponding monomer units (Figure S3). This was observed for both the WT and A53E αSyn dimer, therefore supporting the idea that both dimers exist as noncovalently bound complexes.

**Figure 2:**
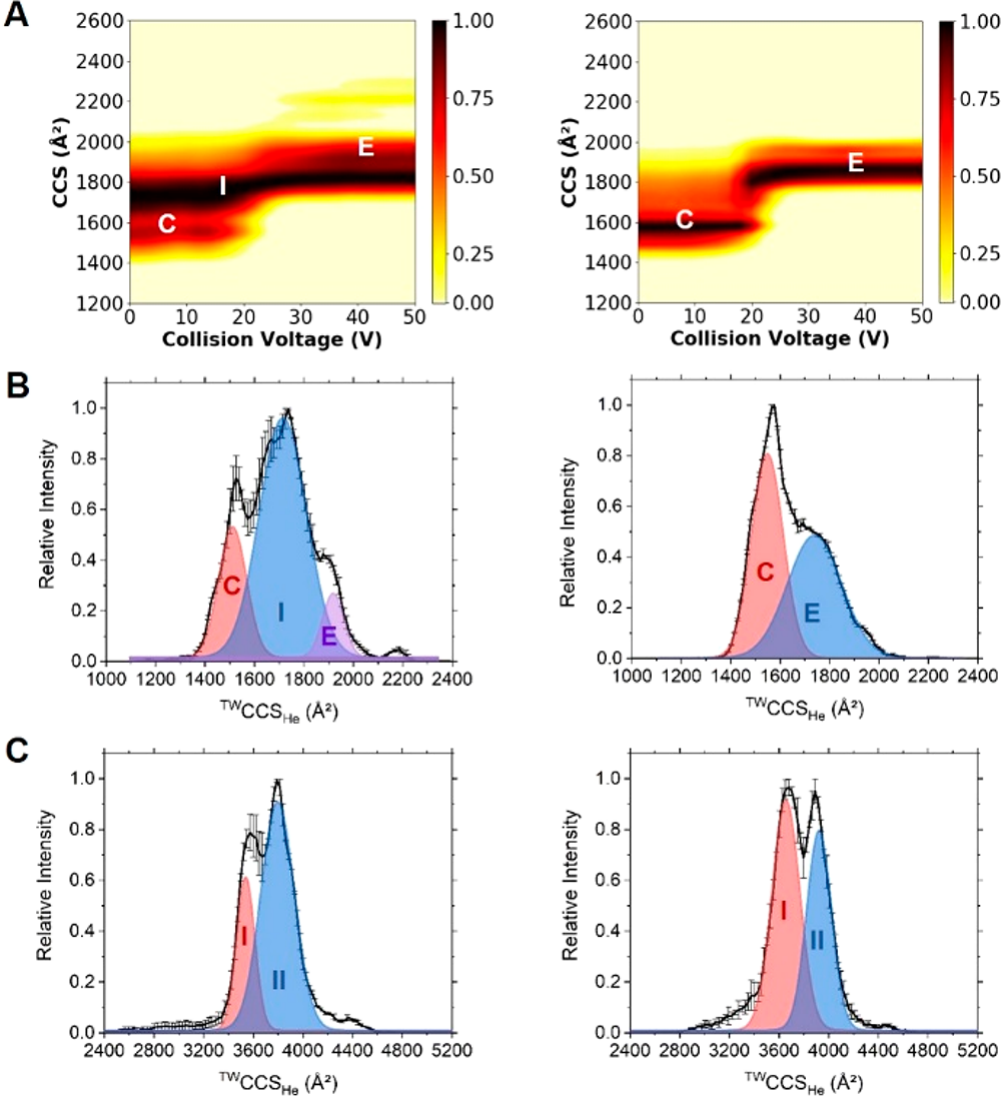
Native ion mobility-mass spectrometry of WT (left) and A53E (right) αSyn. (A) Ion mobility collision induced unfolding profile of the [M+7H]^7+^ monomer species, showing compact (C), intermediate (I), and extended (E) conformational families. (B) Collision cross section distribution of the [M+7H]^7+^ monomer species at a trap voltage of 10 V, representing low preactivation conformations of the protein. (C) Collision cross-section distribution of the [2M+15H]^15+^ dimer species at a trap voltage of 10 V, representing two low preactivation voltage conformational families of the protein, I and II.

Ion mobility mass spectrometry (IM-MS) is a commonly used technique for determining differences in both gas phase structure and stability of protein variants associated with disease, including amyloidogenic proteins.^52^ Here, we initially employed this method to investigate potential differences in the gas-phase structure between monomeric WT and A53E αSyn. We used collision-induced unfolding (CIU) to determine differences in stability of the [M+7H]^7+^ charge state of the protein monomer by increasing the trap voltage in 2 V increments prior to ion-mobility analysis. We observed that WT αSyn exhibits a CIU profile comprising three conformational families, compact (C), intermediate (I), and extended (E), and a transition from compact to extended conformation at around 20 V (Figure 2A). In contrast, when analyzing the monomeric A53E αSyn variant, the protein seemed to only exist as two conformational families, compact (C) and extended (E). When comparing the WT and A53E αSyn variant CIU plots, a root-mean-square deviation (RMSD) value of 19.14 was obtained (Figure S5). To further understand the conformational families occupied by the WT and A53E [M+7H]^7+^ monomer, we looked at the collision cross section distributions (CCSDs) with low preactivation voltages applied. The CCSDs clearly show the three conformational families of WT αSyn, centered on, 1512 Å^2^ (compact), 1715 Å^2^ (intermediate), and 1919 Å^2^ (extended), whereas A53E exists in two conformational families centered on 1549 Å^2^ (compact) and 1739 Å^2^ (extended) (Figure 2B and Table S1).

We were unable to apply the same CIU experimental approach to study the dimeric forms of WT and A53E, due to the low abundance of the species resulting in low S/N. However, we were able to compare the ^TW^CCS_He_ profiles of the [2M+15H]^15+^ dimeric species with both low and high preactivation voltages applied. At low preactivation voltage, two distinct conformational families were observed for both WT and A53E dimers (Figure 2C). These conformations are very similar for the two protein variants, with the compact conformation centered on 3539 and 3656 Å^2^ and the extended conformation centered on 3796 and 3923 Å^2^ for the WT and A53E proteins, respectively (full details in Supporting Information, Table S1). When comparing the CCSDs at higher preactivation voltages, we see similar profiles for the two protein variants (Figure S4B). These observations suggest that subtle differences in conformation and stability may exist between WT and A53E monomers; however, the dimeric forms of these two variants display similar mobility profiles, suggesting the possibility of a common orientation and dimer interaction in αSyn variants.

While this IM-MS data provides some global understanding of the overall conformational families that are occupied by the dimeric species of αSyn, it does not provide enough resolution to suggest the potential binding interface of the dimer. To investigate this further, we chose to employ native electron capture dissociation (ECD) mass spectrometry.

### Isotope Depletion Enables the Production of αSyn Variants with Reduced ^13^C and ^15^N Content

ECD is an extremely versatile top-down fragmentation technique, owing to its ability to retain labile bonds.^24^ This makes it an ideal method for the localization of protein–ligand binding sites but also the localization of protein–protein interaction sites between protein complexes and higher order protein oligomers.^26^ However, fragments deriving from native top-down experiments are often of low signal-to-noise ratio (S/N), making it difficult to confidently assign these fragment ions. In addition, we observed that the dimeric species of αSyn constitutes only a low percentage of the overall signal in the native CSD of the protein (Figure S1), further compounding the difficulties with fragment assignment. To circumvent these issues, we have integrated isotope depletion mass spectrometry (ID-MS) into our native top-down workflow based on the methodology described previously.^29^

**Figure 3:**
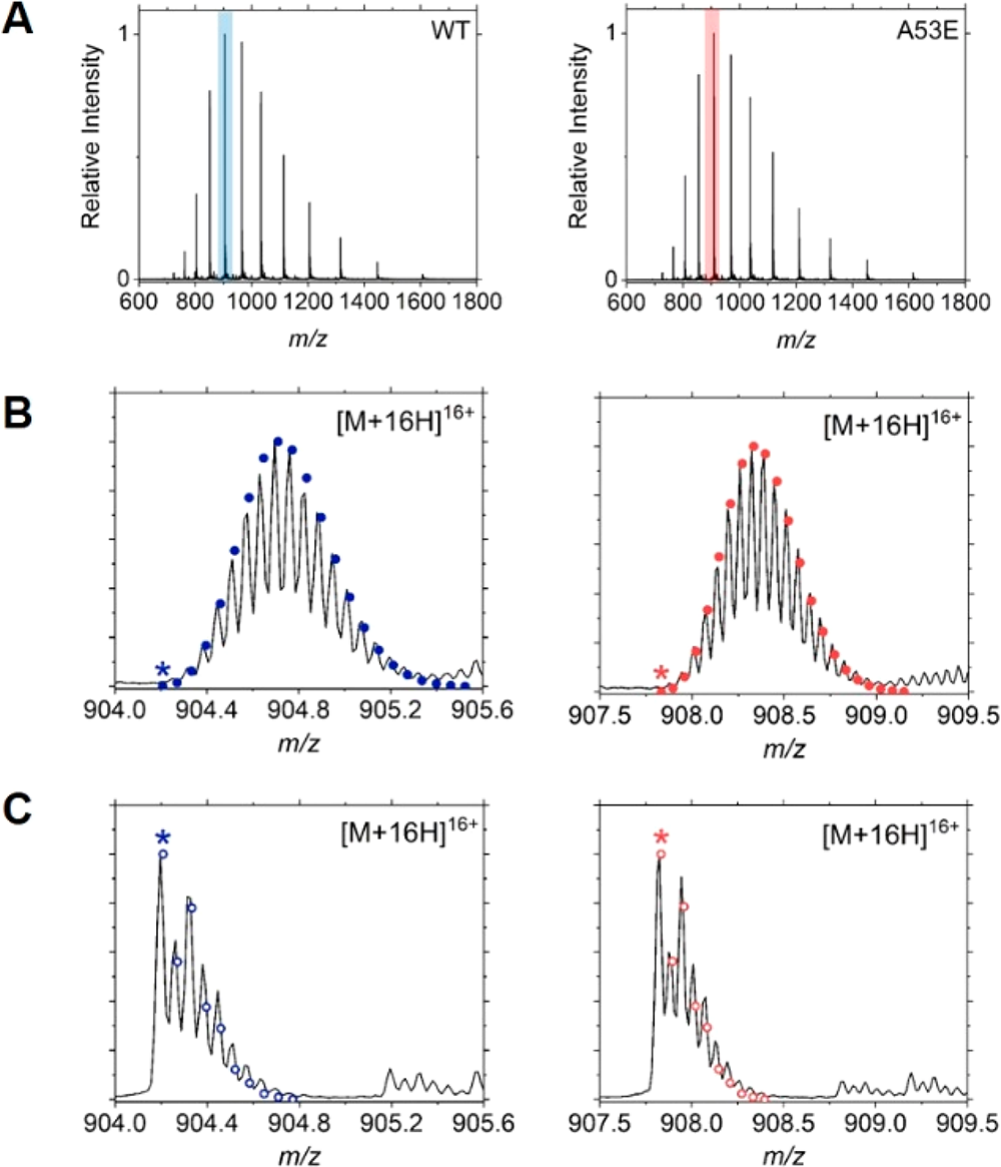
LC–MS analysis of the isotopic distributions of WT (left) and A53E (right) αSyn. (A) The denatured charge state distributions of WT (monoisotopic molecular mass 14,451.2 Da) and A53E (monoisotopic molecular mass 14,509.2 Da), highlighting the [M+16H]^16+^ charge state. (B) The observed isotope distribution of the [M+16H]^16+^ charge state of samples prepared using natural abundance isotope cell media (^12^C 98.89%, ^14^N 99.63%), with the theoretical isotope distribution overlaid as a scatter plot. (C) The observed isotope distribution of the [M+16H]^16+^ charge state of samples prepared using isotopically depleted cell media (^12^C 99.95%, ^14^N 99.99%), with the theoretical isotope distribution overlaid as a scatter plot. The monoisotopic signal is annotated with an asterisk (*).

αSyn protein samples prepared using natural abundance C- and N-isotope media and isotopically depleted media were analyzed via LC–MS on a quadrupole time-of-flight (qTOF) instrument for intact mass determination. The proteins demonstrate identical CSDs (Figure 3A); however, the isotopologue distributions of the ID protein samples displayed reduced width and prominent monoisotopic signals (Figure 3C, marked with an asterisk), characteristic of ID-protein samples. For both WT and A53E, this method results in consistent, reproducible production of protein with isotopic composition consistent with 99.95% ^12^C and 99.99% ^14^N, as seen in previous studies.^29,53^

### Native Top-Down ECD of the αSyn Dimer Displays Reduced Fragmentation in Comparison to the αSyn Monomer

**Figure 4:**
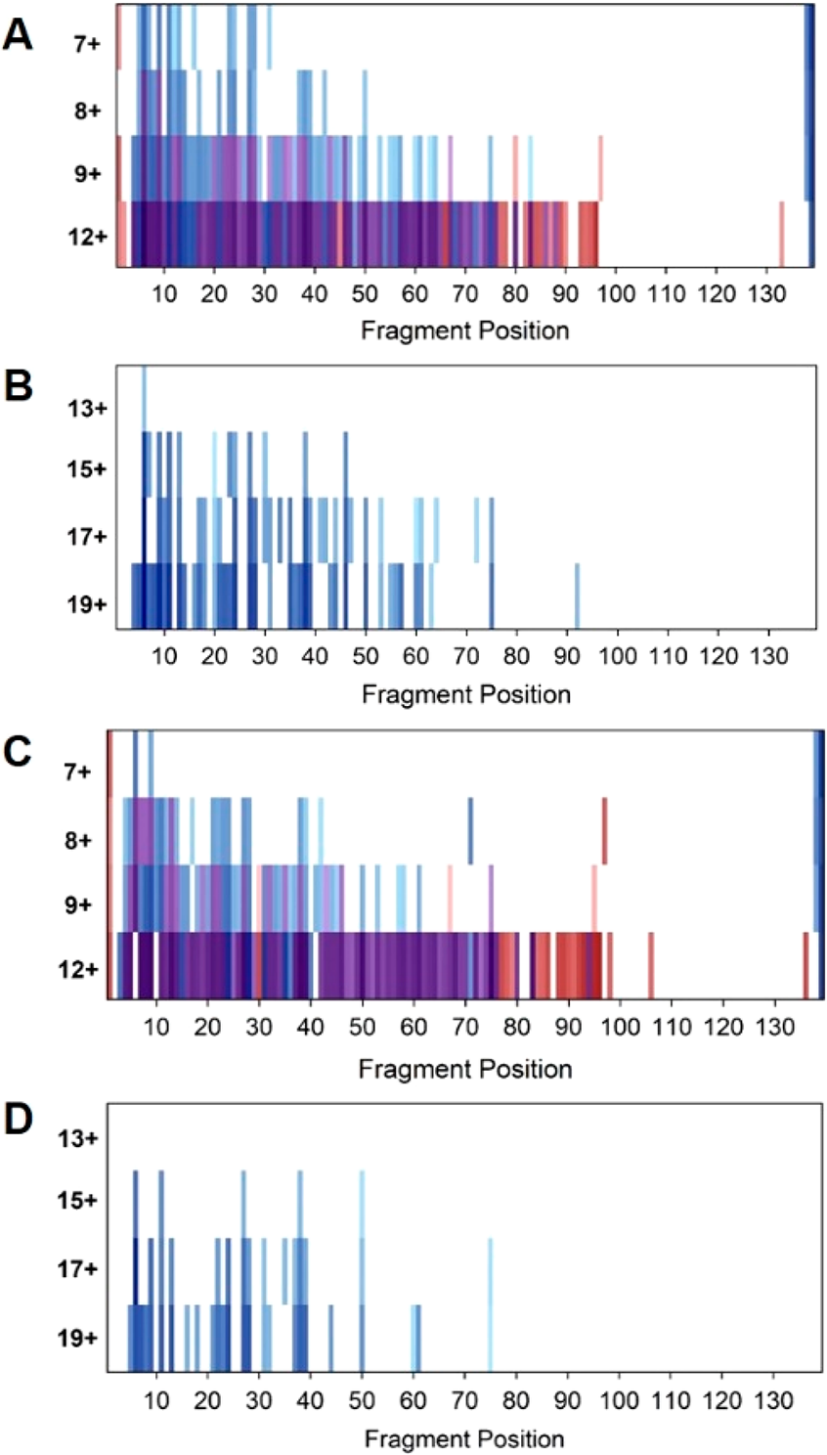
Heatmaps showing native top-down ECD of increasing αSyn charge states. ECD fragments are plotted as position of fragmentation along the sequence. (A) ECD of WT monomer. (B) ECD of WT dimer. (C) ECD of A53E monomer. (D) ECD of A53E dimer. Heatmaps were generated by normalizing the intensity of individual fragments by charge and to the intensity of the precursor ion within the spectrum. Intensities are plotted on a log scale. Blue = *c* ions; red = *z* ions; purple = complementary fragmentation.

Native top-down mass spectrometry was performed on individual charge states of both the monomer and dimer αSyn species using ECD. Initially, several low charge states of the monomer ([M+7H]^7+^ to [M+9H]^9+^) were chosen, to correlate information with IM-MS CIU experiments, in addition to a representative higher charge state, [M+12H]^12+^ (Figure 4A). ECD of the isolated [M+7H]^7+^ and [M+8H]^8+^ charge states resulted in limited fragmentation, with only 15 and 22 N-terminal *c* ions observed, respectively, deriving from a handful of cleavages in the N-terminal domain. However, ECD of charge states with a higher precursor charge ([M+9H]^9+^ and [M+12H]^12+^) resulted in more extensive fragmentation in both the N-terminal and NAC regions of the protein and both *c* and *z* ions were observed ([M+12H]^12+^ N-terminus = 58 *c* ions, 47 *z* ions, NAC region = 19 *c* ions, 28 *z* ions). Interestingly, there was a notable lack of fragmentation in the C-terminal region of the protein, perhaps due to the charge partitioning of the αSyn amino acid sequence, as the C-terminus of the protein is highly acidic in comparison to the basic N-terminus.

Overall, the patterns of fragmentation between the WT and A53E αSyn monomer are highly similar (Figure 4A,C). However, it was noted that A53E exhibits a lower rate of fragmentation throughout the protein, with 72% less fragment ions identified in the [M+7H]^7+^ charge state. This correlates with the CCSDs identified from IM-MS experiments, which revealed that the A53E monomer favors a more compact conformation with a lower ^TW^CCS_He_ than that of WT αSyn.

Following this, we isolated charge states of dimeric αSyn and subjected them to ECD under the same experimental conditions. Charge states unique to the dimer species and of similar charge density to the analyzed monomer were chosen, i.e., from [2M+13H]^13+^ to [2M+19H]^19+^. In our initial analyses of these spectra, we assigned only monomeric *c*- and *z*-fragment ions (i.e., fragments that did not retain noncovalent association between the two αSyn monomeric units, here termed apo fragment ions). Surprisingly, across all the charge states analyzed we did not assign any apo *z* fragment ions; all observed apo fragment ions were N-terminal *c* ions (Figure 4B,D). Similar to our observations with monomeric αSyn, the number of observed fragments increases with charge state; fragmentation is limited to the N-terminal region at low charge state, while fragmentation in the NAC region is observed when higher charge states are subjected to ECD (WT [2M+19H]^19+^ N-terminus = 30 *c* ions, NAC region = 4 *c* ions). However, it is also clear that the overall extent of fragmentation is significantly reduced when comparing the dimeric charge states to the monomeric species. Furthermore, ECD fragmentation of dimeric A53E follows a similar pattern to WT αSyn (A53E [2M+19H]^19+^ N-terminus = 19 *c* ions, NAC region = 2 *c* ions), albeit displaying a slightly lower overall extent of fragmentation than the WT dimer (Figure 4B,D). Thus, these observations suggest WT and A53E dimeric species share a similar overall topology.

**Figure 5:**
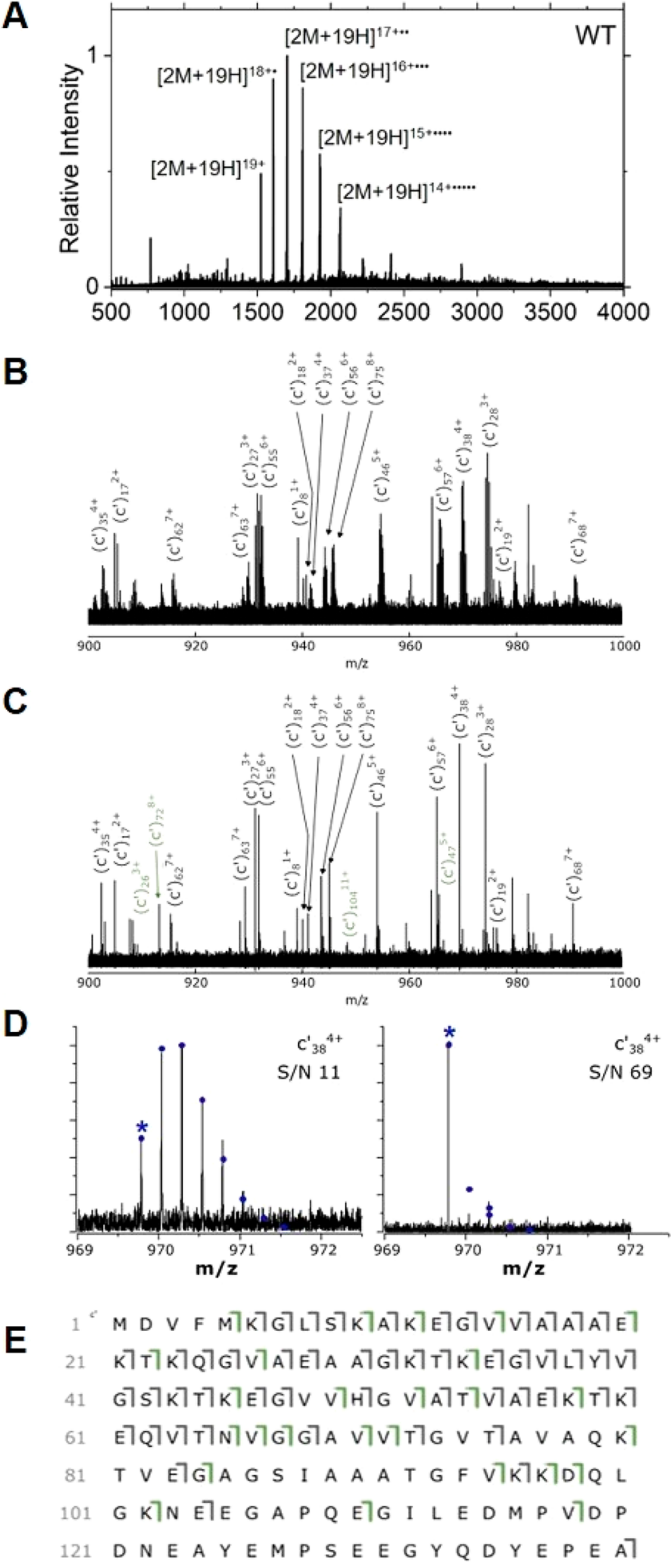
ECD of the WT αSyn dimer produces exclusively apo c-ion fragments . (A) ECD of the [2M+19H]^19+^ charge state of WT αSyn. (B) A 100 *m*/*z* region of the ECD fragmentation spectra for natural abundance and (C) isotopically depleted αSyn. Ions exclusively identified using isotope depletion are colored in green. (D) The *c*_38_^4+^ fragment from isotopically standard protein, S/N 11 (left), and from isotopically depleted protein, S/N 69 (right). The monoisotopic peak is annotated with an asterisk (*). (E) Fragmentation map of [2M+19H]^19+^ dimer fragmentation from isotopically standard WT αSyn (51 apo fragments, 36.7% sequence coverage), and isotopically depleted WT αSyn (77 apo fragments, 55.4% sequence coverage). Fragments unique to the isotopically depleted protein are shown in green.

An example native top-down fragmentation mass spectrum of the WT αSyn dimer is shown in Figure 5, where the [2M+19H]^19+^ species was isolated and subjected to ECD in the ICR cell. Data were collected for both the isotopically standard protein and the isotopically depleted protein under identical experimental conditions. ECD of this dimer charge state resulted in a significant amount of electron capture without dissociation of the complex (ECnoD) (Figure 5A), and the accompanying fragment ions exhibited low S/N, a common phenomenon in native top-down ECD experiments.^54^ For isotopically standard WT [2M+19H]^19+^ dimer, ECD fragmentation resulted in 51 assignable apo fragment ions, which represents 36.7% sequence coverage of the protein (Figure 5E). As stated above, every assigned apo fragment was a *c* ion; this was confirmed by manual analysis of the fragmentation spectrum in addition to peak assignment in AutoVectis and ProSight Lite. In comparison, analysis of the resulting ECD fragmentation spectrum from isotopically depleted WT [2M+19H]^19+^ dimer yielded 77 assignable apo fragment ions, representing 55.4% protein sequence coverage (Figure 5E); again, these assignments were also entirely *c* ions.

We have previously reported the increase in assignable top-down fragment ions as a result of isotope depletion in top-down fragmentation experiments under denaturing conditions.^29^ However, we now demonstrate for the first time that this technique also increases sequence coverage in native top-down experiments. This is demonstrated in Figures 5A,B, which show a 100 *m*/*z* region of a fragmentation spectrum for both isotopically standard and depleted protein samples. In this stretch of the isotopically standard data, 17 *c* ions were identified with a S/N between 11 and 25 (Figure 5C). In that same spectral region, for the isotopically depleted protein, 21 *c* ions were observed with an S/N between 16 and 69 (Figure 5D).

Isotope depletion results in the ion signal being spread over fewer isotopologues, resulting in the observed increase in S:N for an individual fragment; in addition, the decrease in spectral complexity enables overlapping fragment ion isotope distributions to be more easily distinguished and separated. In the isotopically standard data, the overall S/N of the *c*_38_^4+^ ion was 11 over 6 isotopologue peaks (visible above the noise). In the depleted data, the same fragment ion exhibited a S/N of 69 over 3 isotopologue peaks, representing a 6-fold increase in S/N. In both instances the monoisotopic peak is visible, but it is significantly greater in the isotopically depleted spectrum. This observation was also noted in the analysis of the A53E αSyn variant, with substantially more fragment ions assigned using the isotope depletion strategy (see the Supporting Information; Figure S8).

### ECD Fragments Unique to the αSyn Dimer Indicate the Location of the Complex Interface

The striking feature of our native top-down ECD study of the αSyn dimer is the production of exclusively apo*c* ion fragments. We postulate that this observation could result from the dimer interface of the complex forming via interactions located somewhere in the C-terminal region of both monomer units. This C-terminus domain to C-terminus domain interaction between monomer pairs would inhibit fragmentation in the C-terminal region of both monomer units. Furthermore, any C-terminal fragments would likely retain the interaction with the other monomer unit and thus not present as apo *z*-ions. To support this hypothesis, we analyzed the top-down ECD fragmentation data sets in an effort to assign fragment ions which retain the noncovalent dimer interface, i.e., a *c*- or *z*- fragment ion still associated with an intact αSyn monomer unit (here termed holo fragment ions). These theoretical fragment ions all have a molecular mass higher than a monomer of αSyn and are in the form [M·*c*+*n*H]^*n*+^ or [M·*z*+*n*H]^*n*+^.

**Figure 6:**
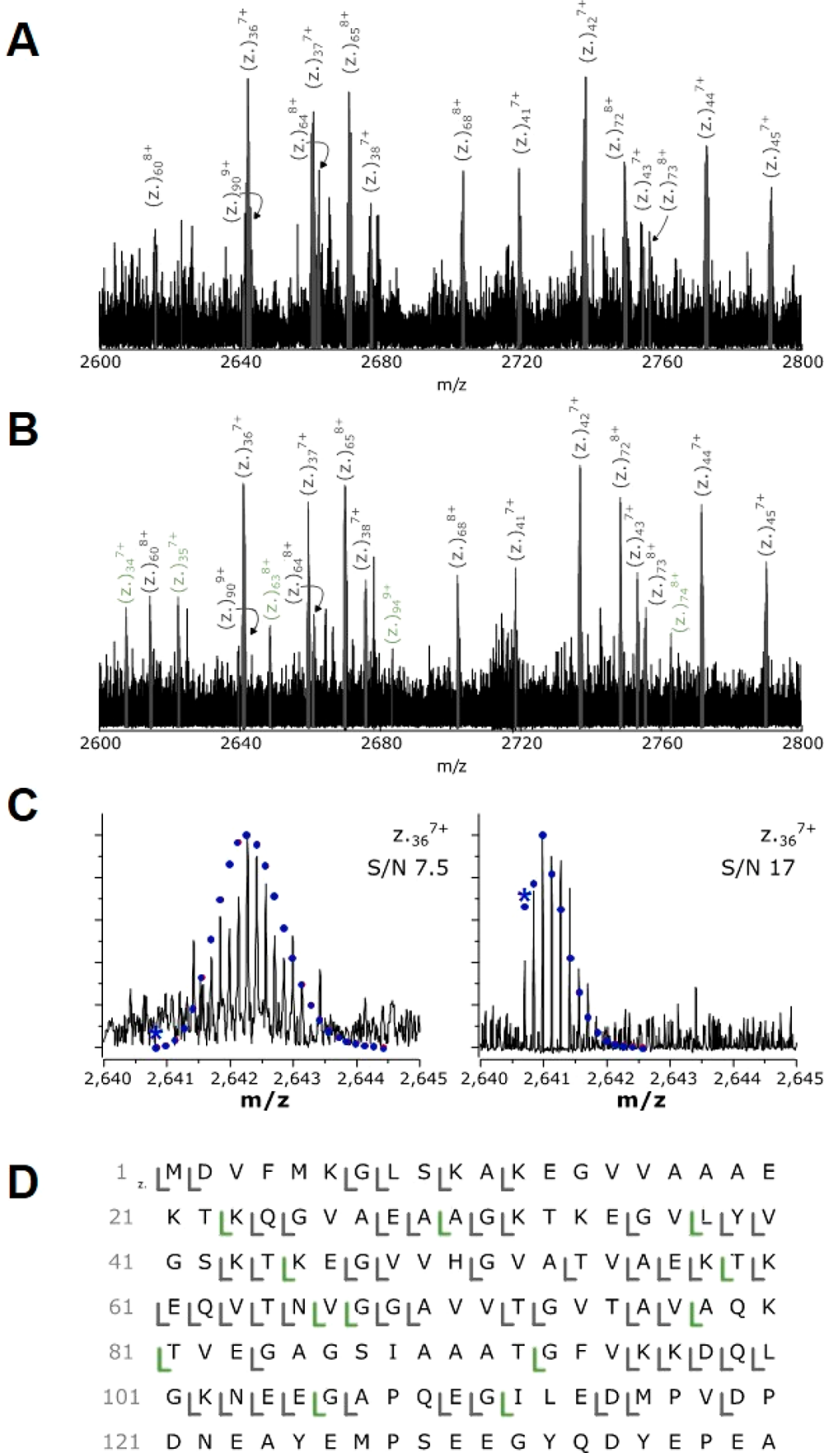
ECD of the WT αSyn dimer produces exclusively holo z-ion fragments . (A) A 200 *m*/*z* region of the ECD fragmentation spectra for natural abundance and (B) isotopically depleted αSyn. Ions exclusively identified using isotope depletion are shown in green. (C) The holo *z*_36_^7+^ fragment from isotopically standard protein, S/N 7.5 (left), and from isotopically depleted protein, S/N 17 (right). The monoisotopic peak is annotated with an asterisk (*). (D) Fragmentation map of [2M+19H]^19+^ dimer fragmentation from isotopically standard WT αSyn (52 holo fragments, 37.4% sequence coverage) and isotopically depleted WT αSyn (64 holo fragments, 46.0% sequence coverage). Fragments unique to the isotopically depleted protein are shown in green.

These holo fragment ions are of particular interest as they retain the dimer interface and therefore provide information about the location of the noncovalent interface within the dimer assembly. However, these are particularly challenging to confidently identify within the spectra; not only do they derive from a low abundant precursor ion, but due to their larger mass, they have a wider isotopologue distribution over which the signal is spread, thus reducing S/N. Therefore, isotope depletion has the potential to be a powerful strategy for increasing the number of assignments of these holo-fragment ions. Analysis and comparison of the ECD spectra of isotopically standard and isotopically depleted forms of the [2M+19H]^19+^ αSyn dimeric species showed this to be the case. Isotopically standard WT αSyn generated 52 assignable fragment ions, whereas the isotopically depleted spectrum from the same charge state allowed 64 holo fragment ions to be assigned (Figure 6D).

The benefits of isotope depletion are well demonstrated by analysis of the holo *z*_36_^7+^ fragment ion, which was assigned in both protein conditions. In the standard spectrum the fragment exhibited 12 isotopologue peaks and an overall S/N of 7.5 (Figure 6C, left), whereas in the depleted spectrum, the fragment had 8 isotopologue peaks and an overall S/N of 17 (Figure 6C, right). This represents a 2.3-fold increase in S/N. Furthermore, the monoisotopic peak was also clearly visible in the isotopologue distribution, whereas in the isotopically standard data set the monoisotopologue for holo *z*_36_^7+^ is below the noise level of the spectrum, hampering confident monoisotopic mass determination of these low S/N species. It is clear after native top-down ECD of the αSyn dimer that all the holo fragment ions produced were holo *z* ions; i.e., they consisted of a C-terminal *z* ion fragment still associated with an intact αSyn monomer and these ions ranged from the holo *z*_36_ to the holo *z*_139_ ion. This trend was also observed during analysis of the A53E variant, where the isotope depletion method enables significantly more fragment ions to be assigned, and all assigned holo fragment ions were *z* ions (see Supporting Information; Figure S8). A small number of *b* and *y* fragment ions were also present in both the WT and A53E ECD spectra (WT = 1 apo *b*, 1 apo *y*, 4 holo *y*; A53E = 3 apo *y*, 5 holo *y*). We believe these ions originate from slight dissociation of the dimer after isolation in the quadrupole; however, the majority of the dimer species remain noncovalently bound during fragmentation.

**Figure 7:**
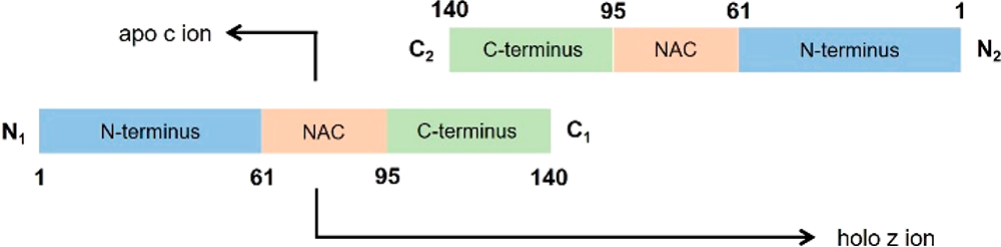
Schematic of the potential dimer interface of αSyn. The proposed interface of the αSyn dimer exists between the two C-termini of both monomer units. The degree of overlap between the monomer units is believed to extend partly into the C-terminal domain of the protein, generating apo *c* fragment ions, derived from a single monomer unit from the dimer, and holo *z* ions, which retain the noncovalent interaction between both monomer units.

Interpreting the information generated by native top-down fragmentation using ECD allows us to infer details about the location of the binding interface of the αSyn dimer. By generating exclusively apo *c* and holo *z* ions, we hypothesize that our data most likely corresponds to a dimer interface that is located between two monomer C-termini. It is unlikely, however, that this interaction exists only between the very C-termini themselves, and so we can infer the degree of overlap between the two monomer units by the position of the ECD cleavages identified. The smallest holo z ion we observed for the WT dimer was the *z*_23_ ion, and the largest apo *c* ion observed was *c*_118_ (Figure S6). These cleavage positions suggest an overlap between the two monomers which includes the C-terminal 23 residues of the protein, i.e., encompassing about half of the acidic C-terminus of αSyn (Figure 7). The observation of a dimer interface that is inclusive of the C-terminus of the protein was also noted for the A53E dimer (Figure S7). This further confirms the finding of a similar CCSD profile for both the WT and A53E dimer (Figure 2C), suggesting that both dimers exhibit similar interface location and overall topology.

This proposed dimer interface is in agreement with what has been observed previously using atomic force microscopy.^55^ These findings suggest that αSyn can form intermolecular interactions between the C-termini of two monomer units, within a dimer species. In addition, a recent hydrogen–deuterium exchange MS investigation observed that peptides primarily within the C-terminus and the NAC domain of the protein are important in the formation of higher order species of αSyn.^56^ Interestingly, none of the identified familial mutations associated with PD occur within this region of the protein.

Together, these observations suggest that the gas-phase stable dimer investigated here may be an off-aggregation pathway endogenous multimer of αSyn. Both structural and dynamic studies of higher order αSyn species highlight the NAC domain as forming the core of the fibrillar structure, with the C-terminal region of the protein remaining flexible and solvent-accessible.^8−12,57−59^ Therefore, this naturally occurring dimer, with an interface located toward the C-terminus, may be part of the pool of endogenous oligomers of αSyn with an unknown physiological function.

## Conclusions

This study demonstrates the ability of native mass spectrometry to investigate stable, naturally occurring species of αSyn. We showed that despite the structural heterogeneity of monomeric WT and A53E αSyn in the gas-phase, the ion mobility profiles of the dimeric species indicate a more common overall conformation shared between WT and A53E. This similarity was also highlighted by native top-down fragmentation using ECD, with both dimers demonstrating similar fragmentation efficiency and positioning. Using this data, we were able to infer the location of the interface for the naturally occurring αSyn dimer, which represents the first time residue-level information has been provided for this αSyn oligomer. Furthermore, we demonstrate the first use of our novel isotope depletion method for increasing the efficiency of fragment assignment for native top-down studies. Here, we have shown that this method significantly increases the S/N of low-abundant native fragment ions (up to 6-fold) and enables the confident identification of further fragments. This therefore demonstrates the scope of this method for improving top-down sequence coverage in native studies.

## Data Availability

The data sets used in this study can be found on Edinburgh DataShare using the following DOI: 10.7488/ds/3811.

## Supporting Information Available

Supplementary experimental information including details of primers used for site-directed mutagenesis; thioflavin T fluorescence spectra; collision-induced dissociation analysis of the αSyn dimer; further CCSD analysis of monomeric and dimeric αSyn; native top-down fragmentation using ECD of the A53E dimer; ECD fragment assignment lists (PDF)
