# Supplementary material for: Determining the Location of the α-Synuclein Dimer Interface Using Native Top-Down Fragmentation and Isotope Depletion-Mass Spectrometry
Source: J Am Soc Mass Spectrom. 2023 Mar 28;34(5):847–56. doi: 10.1021/jasms.2c00339 (PMC10161212; doi:10.1021/jasms.2c00339)
Supplement: Supplementary file 1 — js2c00339_si_001.pdf [file js2c00339_si_001.pdf]

Supporting Information

for

Determining the Location of the Alpha-Synuclein Dimer Interface using Native Top-Down Fragmentation and Isotope Depletion-Mass Spectrometry

Kiani Jeacock[1], Alexandre Chappard[1], Kelly J. Gallagher[1], C. Logan Mackay[1], David P. A. Kilgour[2], Mathew H. Horrocks[1], Tilo Kunath[3] and David J. Clarke[1]*

[1]The EastCHEM School of Chemistry, University of Edinburgh. Edinburgh, EH9 3FJ, UK

[2]Chemistry and Forensics, Nottingham Trent University, Nottingham, NG11 8NS, UK

[3]Centre for Regenerative Medicine, Institute for Stem Cell Research, University of Edinburgh, Edinburgh, EH16 4UU, UK

*Corresponding author – David J. Clarke, dave.clarke@ed.ac.uk

Table of Contents

Experimental Details ... 3

Primers used for site-directed mutagenesis of αSyn ... 3

Thioflavin T (ThT) Fluorescence ... 3

Native Top-Down Fragmentation with Collision Induced Dissociation ... 3

Figure S1. Native mass spectrometry of αSyn variants ... 4

Figure S2. Thioflavin T (ThT) fluorescence spectra of αSyn variants ... 5

Figure S3. Native top-down fragmentation by collision induced dissociation (CID) ... 6

Figure S4. Native ion mobility-mass spectrometry of WT (left) and A53E (right) αSyn ... 7

Figure S5. Comparative CIU profiles of WT and A53E αSyn ... 8

Table S1. CCSD assignments for the $[M+7H]^{7+}$ monomer and $[2M+15H]^{15+}$ dimer of WT and A53E αSyn at low and high pre-activation voltages ... 9

Figure S6. Fragmentation map of the WT αSyn dimer ... 10

Figure S7. Fragmentation map of the A53E αSyn dimer ... 11

Figure S8. Enalrged version of Figure 5 in the main text for easier interpretation. ... 12

Figure S9. Enalrged version of Figure 6 in the main text for easier interpretation. ... 13

Table S2. Electron capture dissociation (ECD) fragment assignment list for isotopically standard WT monomer, $[M+12H]^{12+}$ charge state, processed and analyzed using DataAnalysis v4.2 and ProSight Lite ... 14

Table S3. Electron capture dissociation (ECD) fragment assignment list for isotopically standard A53E monomer, $[M+12H]^{12+}$ charge state, processed and analyzed using DataAnalysis v4.2 and ProSight Lite ... 18

Table S4. Electron capture dissociation (ECD) fragment assignment list for isotopically standard WT dimer, $[2M+19H]^{19+}$ charge state, processed and analyzed using DataAnalysis v4.2 and ProSight Lite ... 22

Table S5. Electron capture dissociation (ECD) fragment assignment list for isotopically standard A53E dimer, $[2M+19H]^{19+}$ charge state, processed and analyzed using DataAnalysis v4.2 and ProSight Lite ... 24

Experimental Details

Primers used for site-directed mutagenesis of αSyn

Variant	Primer sequence	
A53E	Forward	5'-cttctcagccactgtttccacaccatgcaccac-3'
	Reverse	5'-gtggtgcatggtgtggaaacagtggctgagaag-3'

Thioflavin T (ThT) Fluorescence

Fluorescence emission of WT and A53E αSyn was monitored using a Synergy HTX Multi-Mode Microplate Reader (BioTek). 100 μM samples were incubated at 37 °C in 96-well, black, clear bottom microplates with 20 μM Thioflavin T (ThT), and a single 3 mm soda-lime glass bead per well. Measurements were taken every 15 minutes, following 30 seconds of orbital shaking at 548 cpm, using excitation filter 460/40 nm, and emission filter 518/20 nm. All reactions were carried out in triplicate. Data was processed in Origin 2019.

Native Top-Down Fragmentation with Collision Induced Dissociation

Samples were prepared identically as for native top-down fragmentation with ECD. Spectra were acquired using a SolariX FT-ICR instrument equipped with an Infinity ICR cell and a 12 T magnet (Bruker Daltonics). Prior to fragmentation, non-mass concurrent charge states of the dimer were isolated in the mass resolving quadrupole. Collision induced dissociation (CID) was conducted using a voltage of 20 V. Data was processed using DataAnalysis v4.2 (Bruker Daltonics).

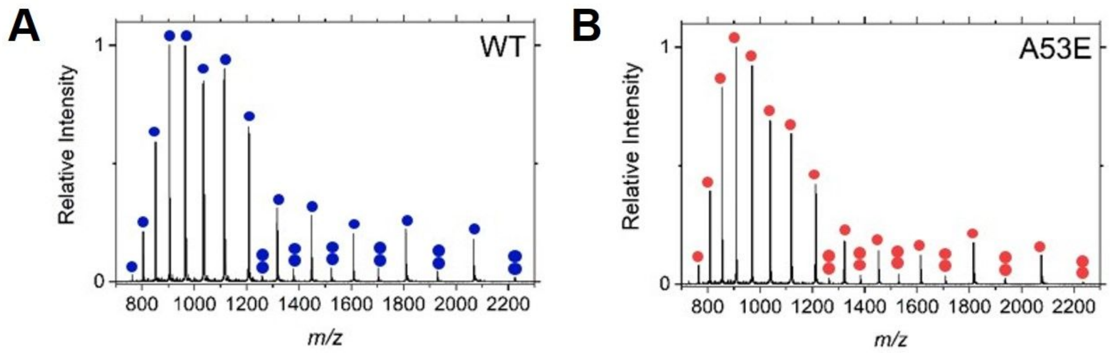

Figure S1. Native mass spectrometry of αSyn variants. Native charge state distribution of (A) WT and (B) A53E αSyn, highlighting both monomeric species in charge states from $[M+7H]^{7+}$ to $[M+19H]^{19+}$ (single circles), and dimeric species in charge states from $[2M+13H]^{13+}$ to $[2M+21]^{21+}$ (double circles).

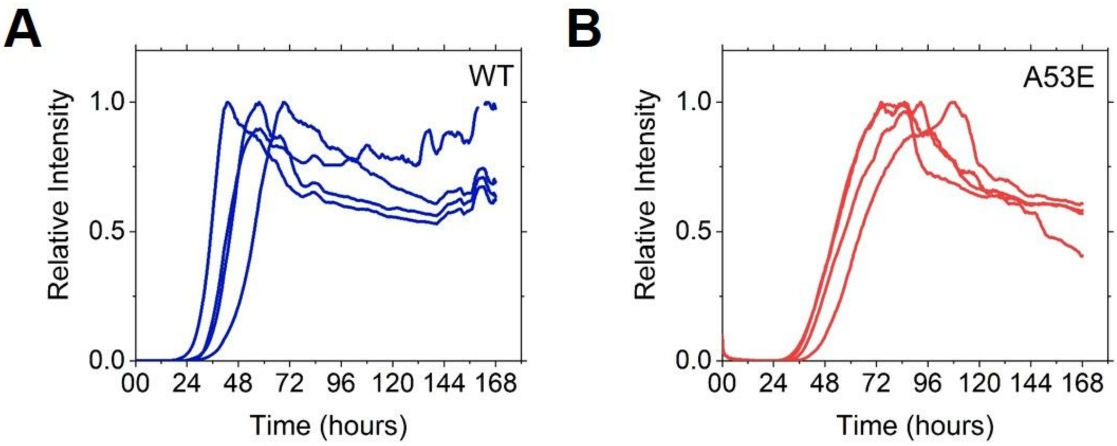

Figure S2. Thioflavin T (ThT) fluorescence spectra of αSyn variants. Fluorescence emission of ThT-positive species of αSyn were monitored at 37 °C in 100 mM ammonium acetate. (A) WT and (B) A53E were incubated at 100 μM for a period of 168 hours with intermittent orbital shaking. Spectra shown are replicates of identical conditions, due to the high variability of the assay. Breaks in the spectra are a result of oversaturation of the photon multiplier tube of the microplate reader.

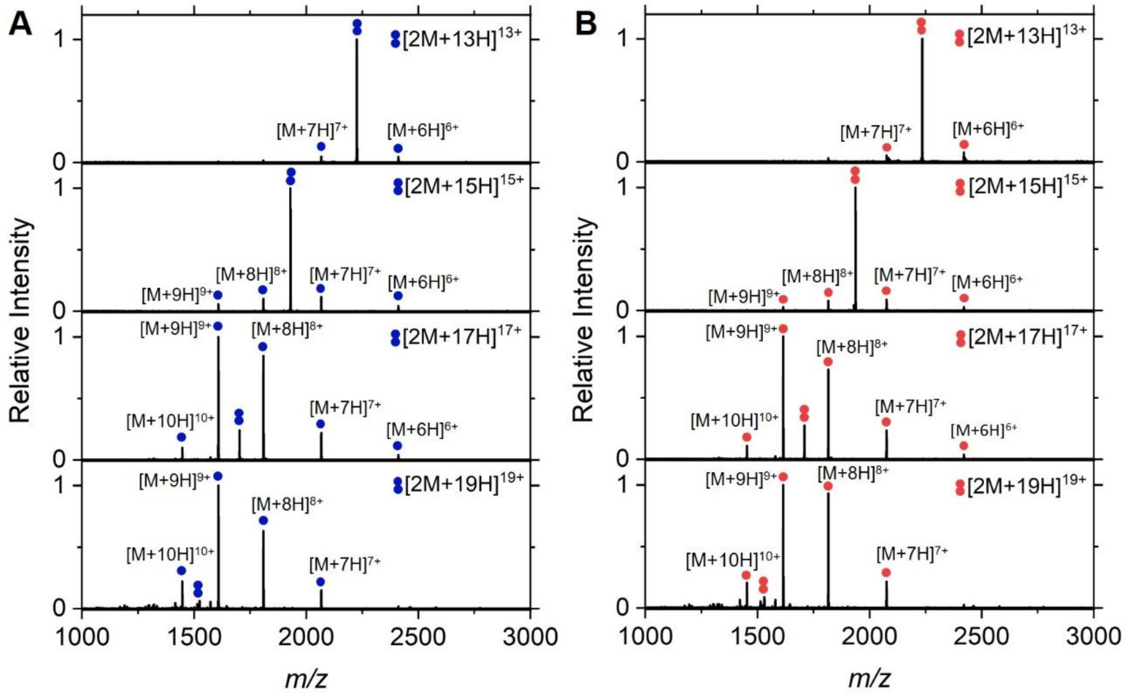

Figure S3. Native top-down fragmentation by collision induced dissociation (CID). CID was conducted at 20 V on multiple charge states of the (A) WT and (B) A53E αSyn dimer, from $[2M+13H]^{13+}$ to $[2M+19H]^{19+}$. In all cases, upon applying CID, the dimer (double circles) dissociated into individual monomer subunits (single circles).

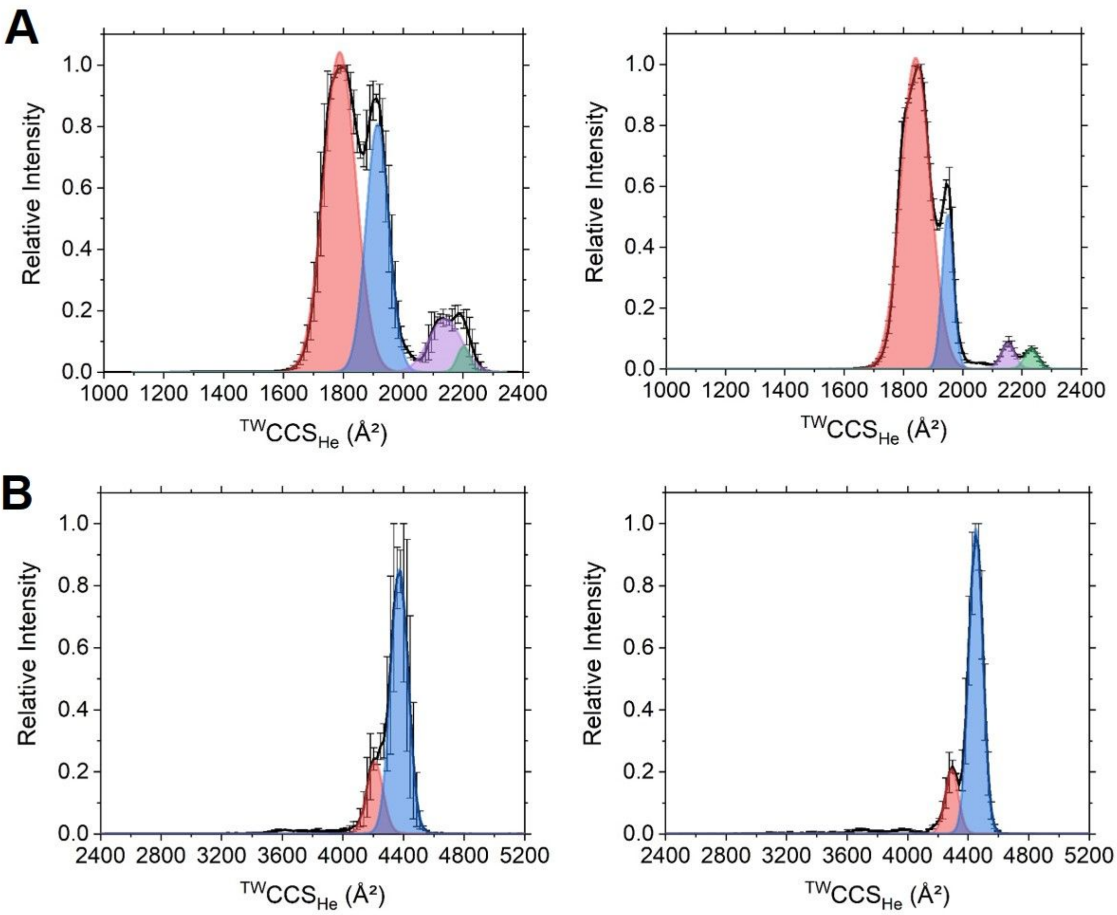

Figure S4. Native ion mobility-mass spectrometry of WT (left) and A53E (right) αSyn. (A) Collision cross section distribution of the $[M+7H]^{7+}$ monomer species at a trap voltage of 40 V, representing high pre-activation conformations of the protein. (B) Collision cross section distribution of the $[2M+15H]^{15+}$ dimer species at a trap voltage of 40 V, again demonstrating the conformations present at high pre-activation voltages.

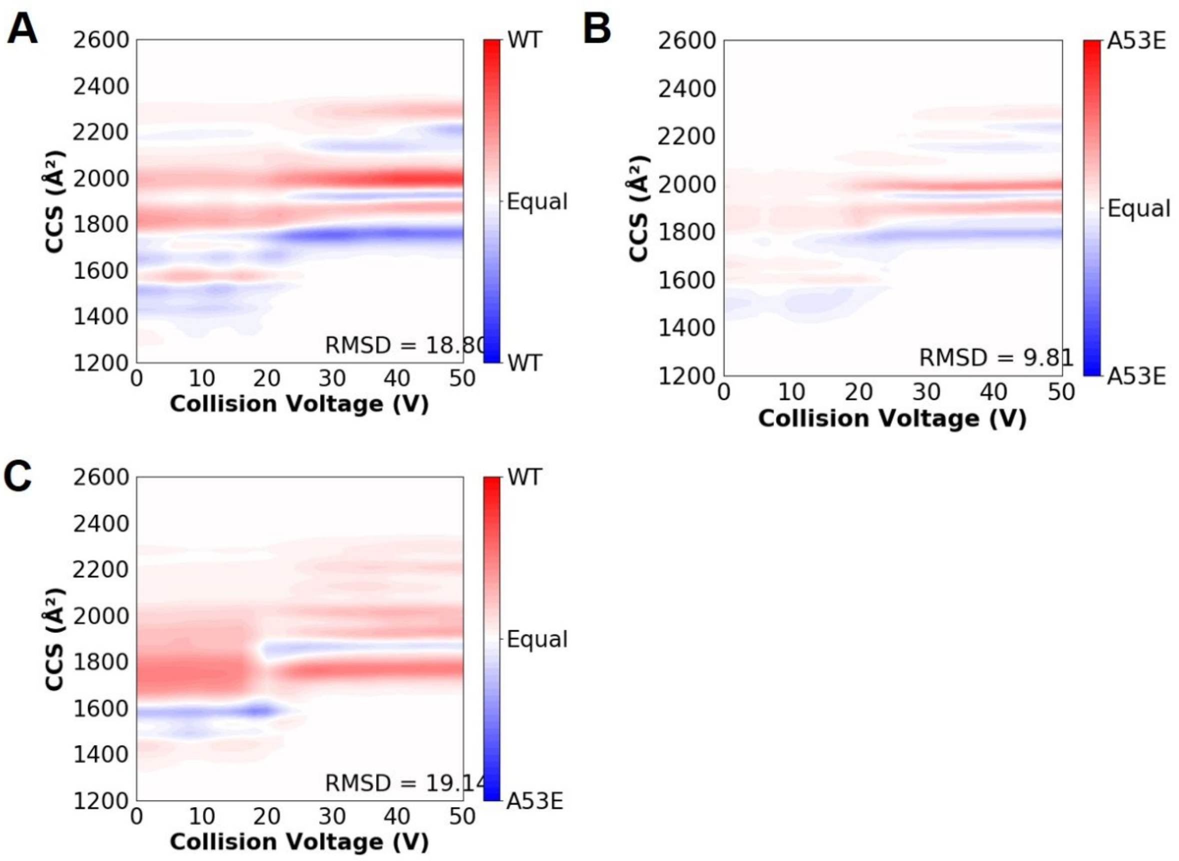

Figure S5. Comparative CIU profiles of WT and A53E αSyn. Comparative profiles of collision induced unfolding (CIU) of the $[M+7H]^{7+}$ monomer of the (**A**) WT αSyn replicates (**B**) A53E αSyn replicates and (**C**) A53E αSyn compared to WT αSyn. The reported root mean square deviation (RMSD) value are: WT vs WT = 18.80, A53E vs A53E = 9.81, WT vs A53E = 19.14.

Table S1. CCSD assignments for the $[M+7H]^{7+}$ monomer and $[2M+15H]^{15+}$ dimer of WT and A53E αSyn at low and high pre-activation voltages.

		**Peak 1**		**Peak 2**		**Peak 3**		**Peak 4**	
		**$^{TW}CCS_{He}$ (Å^2) ± FWHM**	**Occupancy (%)**	**$^{TW}CCS_{He}$ (Å^2) ± FWHM**	**Occupancy (%)**	**$^{TW}CCS_{He}$ (Å^2) ± FWHM**	**Occupancy (%)**	**$^{TW}CCS_{He}$ (Å^2) ± FWHM**	**Occupancy (%)**
**WT monomer**	**10 V**	1512 ± 133	22	1715 ± 226	69	1919 ± 102	9		
	**40 V**	1788 ± 127	57	1914 ± 91	31	2140 ± 130	10	2202 ± 51	2
**A53E monomer**	**10 V**	1549 ± 152	49	1739 ± 259	51				
	**40 V**	1841 ± 118	80	1950 ± 46	15	2152 ± 56	3	2231 ± 54	2
**WT dimer**	**10 V**	3539 ± 174	27	3796 ± 319	73				
	**40 V**	4211 ± 128	21	4373 ± 136	79				
**A53E dimer**	**10 V**	3656 ± 266	56	3923 ± 236	44				
	**40 V**	4291 ± 103	17	4451 ± 109	83				

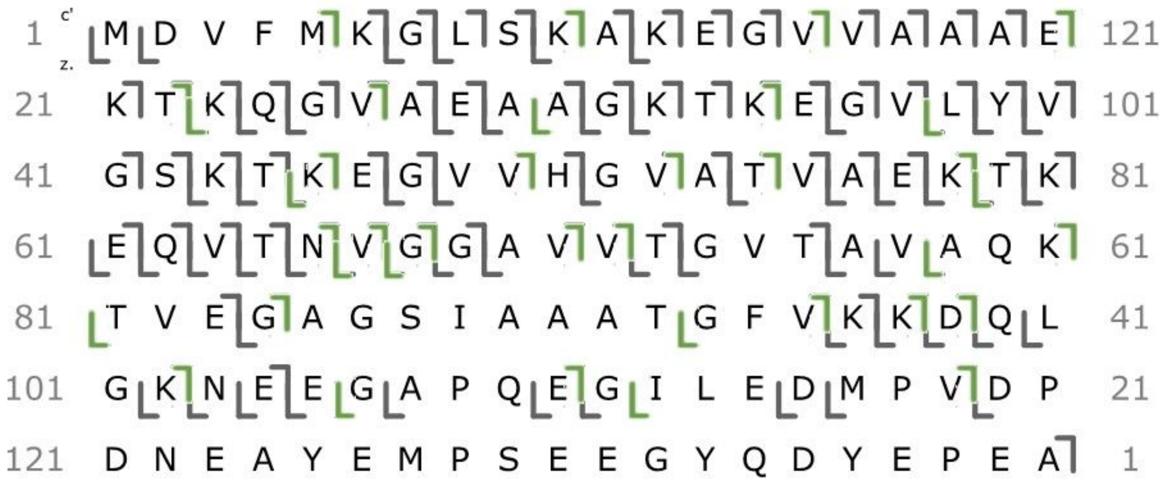

Figure S6. Fragmentation map of the WT αSyn dimer. Fragmentation map of the $[2M+19H]^{19+}$ WT αSyn dimer showing apo *c* ions and holo *z* ions. A total of 141 fragments were identified for the isotopically depleted protein. Fragments unique to the isotopically depleted dimer are shown in green.

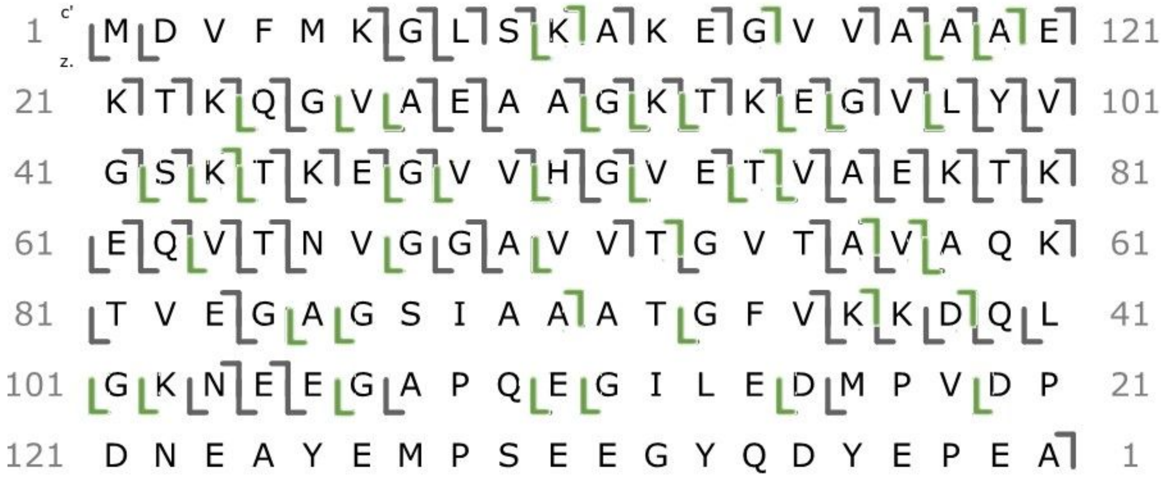

Figure S7. Fragmentation map of the A53E αSyn dimer. Fragmentation map of the $[2M+19H]^{19+}$ A53E αSyn dimer showing apo *c* ions and holo *z* ions. A total of 139 fragments were identified for the isotopically depleted protein. Fragments unique to the isotopically depleted dimer are shown in green.

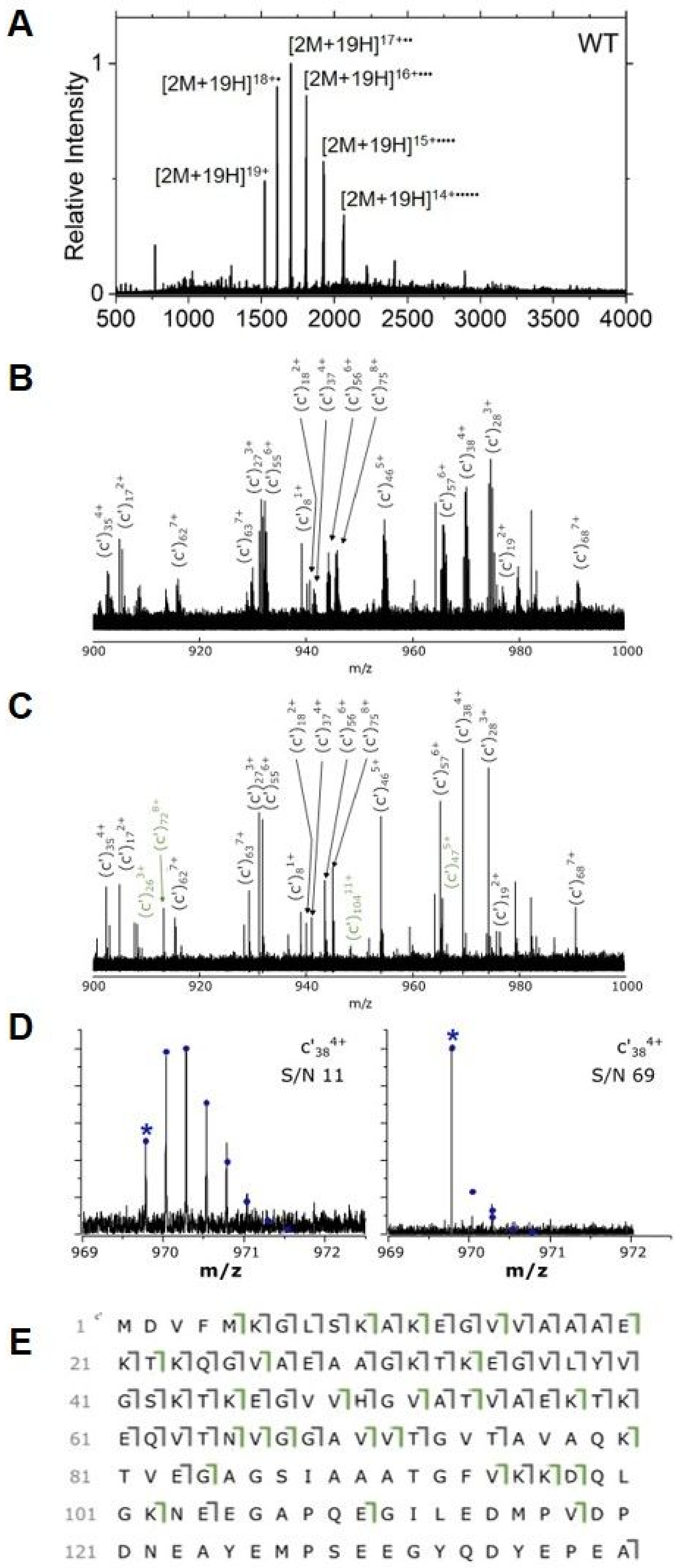

Figure S8. Enlarged version of Figure 5 in the main text for easier interpretation.

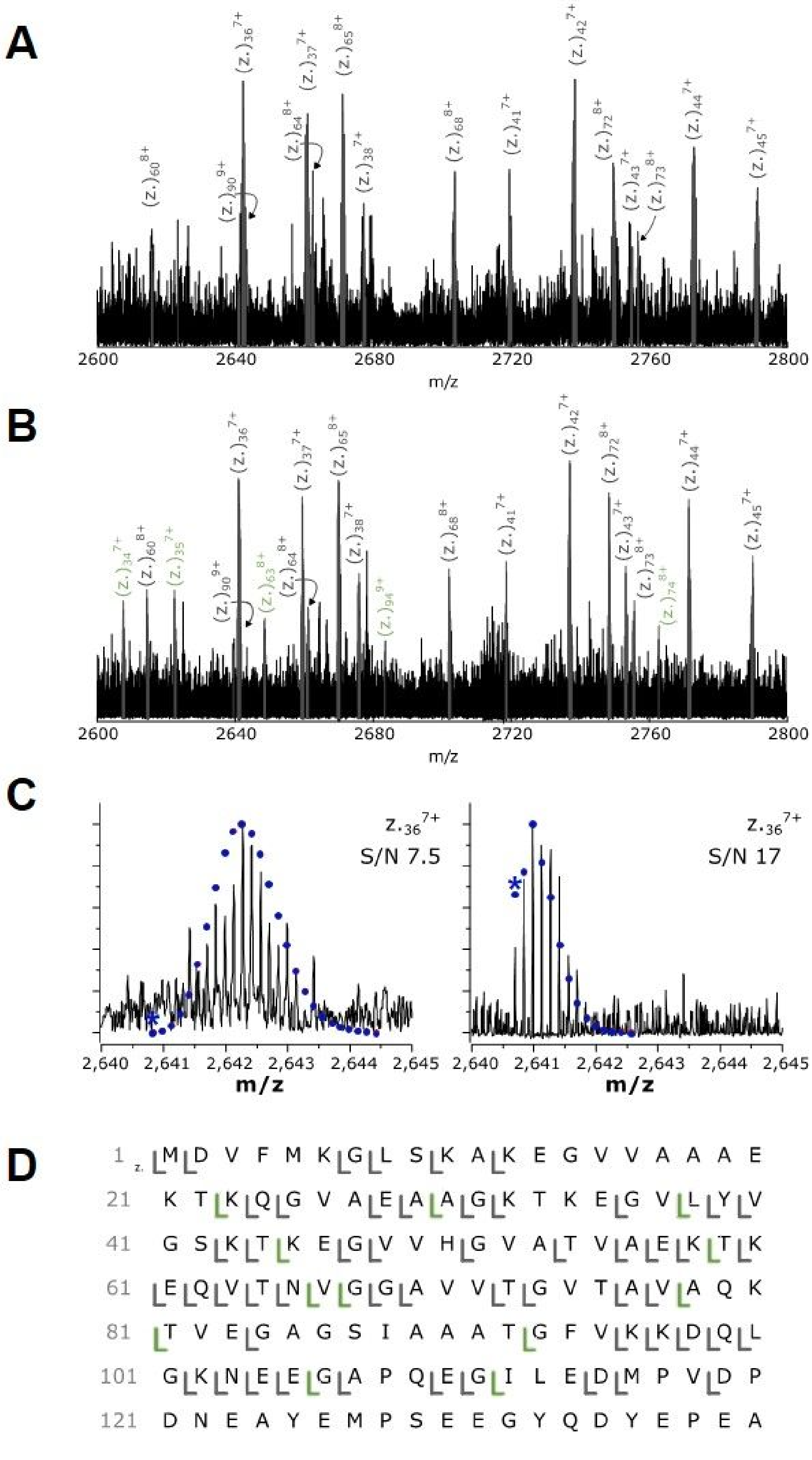

Figure S9. Enlarged version of Figure 6 in the main text for easier interpretation.

Table S2. Electron capture dissociation (ECD) fragment assignment list for isotopically standard WT monomer, $[M+12H]^{12+}$ charge state, processed and analyzed using DataAnalysis v4.2 and ProSight Lite.

Assigned Ion	Theoretical Neutral Mass (Da)	Observed Neutral Mass (Da)	Mass Error (ppm)
C4	509.23055	509.2307235	0.340775156
C5	640.27104	640.2712235	0.286649104
C6	768.366	768.3663635	0.473124943
C7	825.38746	825.3877135	0.307168611
C8	938.47152	938.4719635	0.472612232
C9	1025.50355	1025.504464	0.890814195
C11	1224.63562	1224.636994	1.121585145
C12	1352.73058	1352.732034	1.074517825
C13	1481.77317	1481.775584	1.628814159
C14	1538.79463	1538.796564	1.256524479
C15	1637.86304	1637.855054	-4.876150621
C16	1736.93145	1736.933084	0.94047069
C17	1807.96856	1807.969924	0.754179663
C18	1879.00567	1879.007184	0.805496835
C19	1950.04278	1950.045844	1.57100816
C20	2079.08537	2079.087444	0.997329475
C21	2207.18033	2207.182244	0.866958216
C22	2308.22801	2308.231854	1.665144476
C23	2436.32297	2436.325454	1.019377624
C24	2564.38155	2564.384454	1.132254723
C26	2720.47142	2720.473704	0.839388756
C27	2791.50853	2791.510954	0.868180446
C28	2920.55112	2920.554394	1.120861435
C29	2991.58823	2991.590644	0.80677317
C31	3119.6468	3119.649784	0.956368881
C32	3247.74176	3247.746814	1.556014454
C33	3348.78944	3348.796394	2.07643187
C34	3476.8844	3476.887764	0.967398606
C35	3605.92699	3605.930314	0.921686193
C36	3662.94845	3662.953784	1.45607649
C37	3762.01686	3762.021464	1.22368753
C38	3875.10092	3875.105824	1.265394946
C39	4038.16425	4038.169494	1.298494265
C40	4137.23266	4137.237104	1.074035106

C43	4409.38111	4409.385294	0.948780115
C44	4510.42879	4510.433384	1.018424929
C47	4824.5878	4824.589584	0.369675751
C48	4923.65621	4923.655644	-0.115050048
C49	5022.72462	5022.728424	0.757264912
C50	5159.78353	5159.788004	0.867000155
C52	5315.8734	5315.879104	1.072924934
C53	5386.91051	5386.915994	1.017936554
C55	5587.0266	5587.030264	0.655721439
C56	5658.06371	5658.068974	0.930271094
C57	5787.1063	5787.112114	1.0045665
C58	5915.20126	5915.206624	0.906737215
C59	6016.24894	6016.245074	-0.642670694
C61	6273.38649	6273.392014	0.88047072
C62	6401.44507	6401.448594	0.550427768
C63	6500.51348	6500.518754	0.811248701
C64	6601.56116	6601.565864	0.712488002
C65	6715.60409	6715.608194	0.611044526
C67	6871.69396	6871.698744	0.696121385
C68	6928.71542	6928.720794	0.775545364
C69	6999.75253	6999.757294	0.68052879
C70	7098.82094	7098.828144	1.014750644
C71	7197.88935	7197.893674	0.600666794
C73	7355.95849	7355.963324	0.657090864
C74	7455.0269	7455.036014	1.222468174
C75	7556.07458	7556.081344	0.895112012
C76	7627.11169	7627.116734	0.661263834
C80	8053.37075	8053.378264	0.932967493
C86	8567.60946	8567.613864	0.513974539
C88	8767.72555	8767.730044	0.512508415
C95	9385.04284	9385.048084	0.558711688
Z7	834.3157	834.3226135	8.28647132
Z44	4939.08848	4939.110744	4.507619819
Z46	5166.25185	5166.257474	1.088513159
Z50	5542.42651	5542.435194	1.566738522
Z51	5613.46362	5613.475784	2.166849906
Z52	5684.50073	5684.511704	1.930430418
Z53	5797.58479	5797.598654	2.391260089
Z54	5884.61682	5884.634944	3.079815334
Z55	5941.63828	5941.655254	2.856709264

Z56	6012.67539	6012.693094	2.944368683
Z57	6069.69685	6069.716604	3.254451352
Z58	6198.73944	6198.755884	2.652722102
Z60	6398.85553	6398.875274	3.085478806
Z62	6655.00907	6655.032564	3.53020302
Z64	6825.11459	6825.138954	3.569688508
Z66	6997.19938	6997.237144	5.396949703
Z67	7096.26779	7096.298324	4.302759425
Z69	7254.33693	7254.357744	2.869115857
Z70	7353.40534	7353.430564	3.43018397
Z73	7580.53232	7580.547654	2.0227515
Z74	7637.55378	7637.574204	2.674093526
Z76	7850.66512	7850.688224	2.942875892
Z77	7951.7128	7951.734304	2.704264309
Z78	8050.78121	8050.804224	2.858546583
Z79	8178.83979	8178.866674	3.286961698
Z80	8307.88238	8307.910834	3.424883962
Z81	8435.97734	8435.997474	2.386627217
Z82	8537.02502	8537.052124	3.174821798
Z83	8665.11998	8665.139644	2.269274189
Z85	8865.19968	8865.220254	2.320707245
Z88	9136.35288	9136.377224	2.664469449
Z89	9235.42129	9235.450994	3.216261846
Z90	9292.44275	9292.465804	2.48089052
Z91	9429.50166	9429.524864	2.460738007
Z93	9627.63848	9627.660634	2.301035001
Z95	9813.70253	9813.739934	3.811357947
Z97	10042.84517	10042.8749	2.960668279
Z99	10257.97216	10257.99102	1.838914439
Z100	10314.99362	10315.01797	2.360983828
Z101	10414.06203	10414.08721	2.418223845
Z102	10577.12536	10577.1518	2.500068045
Z103	10690.20942	10690.23661	2.543779271
Z104	10789.27783	10789.30246	2.283149393
Z105	10846.29929	10846.3241	2.28774188
Z108	11204.48452	11204.5007	1.44437998
Z111	11460.63805	11460.67847	3.527162532
Z112	11531.67516	11531.69763	1.948852427
Z113	11660.71775	11660.74266	2.136535131
Z114	11731.75486	11731.77971	2.118483844

Z116	11887.84473	11887.86984	2.11253879
Z118	12143.99827	12144.02257	2.001279363
Z120	12373.14091	12373.17102	2.433782444
Z121	12502.1835	12502.20226	1.500820487
Z122	12573.22061	12573.24465	1.912281179
Z123	12644.25772	12644.28908	2.480456649
Z128	13099.4957	13099.51799	1.701861937
Z130	13298.62777	13298.64486	1.285360672
Z131	13426.72273	13426.75162	2.15194234
Z132	13513.75476	13513.779	1.793989425
Z133	13626.83882	13626.87732	2.825566049
Z134	13683.86028	13683.88776	2.008463443
Z136	13942.99573	13943.00346	0.554653625
Z139	14304.15949	14304.17898	1.362787736

Table S3. Electron capture dissociation (ECD) fragment assignment list for isotopically standard A53E monomer, $[M+12H]^{12+}$ charge state, processed and analyzed using DataAnalysis v4.2 and ProSight Lite.

Assigned Ion	Theoretical Neutral Mass (Da)	Observed Neutral Mass (Da)	Mass Error (ppm)
C4	509.23055	509.2302335	-0.621460908
C5	640.27104	640.2707935	-0.384941477
C6	768.366	768.3662135	0.277905477
C7	825.38746	825.3877235	0.319284134
C8	938.47152	938.4724035	0.941459705
C9	1025.50355	1025.505024	1.43688739
C11	1224.63562	1224.638374	2.248450947
C12	1352.73058	1352.732764	1.614167043
C13	1481.77317	1481.777914	3.201254562
C14	1538.79463	1538.798874	2.75769946
C15	1637.86304	1637.866234	1.949816952
C16	1736.93145	1736.932284	0.479888323
C17	1807.96856	1807.970424	1.030733145
C18	1879.00567	1879.007314	0.874682363
C19	1950.04278	1950.045604	1.447933937
C20	2079.08537	2079.089714	2.089155733
C21	2207.18033	2207.184074	1.696070353
C22	2308.22801	2308.232284	1.851434564
C23	2436.32297	2436.328094	2.1029778
C24	2564.38155	2564.387984	2.508804948
C25	2621.40301	2621.404524	0.577375213
C26	2720.47142	2720.478324	2.537623836
C27	2791.50853	2791.514874	2.272439096
C28	2920.55112	2920.557584	2.213121036
C31	3119.6468	3119.659354	4.024023848
C32	3247.74176	3247.747154	1.66070258
C33	3348.78944	3348.794454	1.497118051
C35	3605.92699	3605.933594	1.831299729
C36	3662.94845	3662.956464	2.187727518
C37	3762.01686	3762.026334	2.518205918
C38	3875.10092	3875.109534	2.222789367
C39	4038.16425	4038.173614	2.318759847
C42	4281.28615	4281.294754	2.00956741
C45	4638.52375	4638.531454	1.660772594

C47	4824.5878	4824.590574	0.574874628
C48	4923.65621	4923.666654	2.121093081
C49	5022.72462	5022.728064	0.685590666
C50	5159.78353	5159.789884	1.231356525
C51	5216.80499	5216.809904	0.941866359
C52	5315.8734	5315.881814	1.582718866
C53	5444.91599	5444.927304	2.077815919
C54	5545.96367	5545.976964	2.396974432
C55	5645.03208	5645.042794	1.897869307
C56	5716.06919	5716.080424	1.965254924
C57	5845.11178	5845.122264	1.793555626
C59	6074.25442	6074.266324	1.959669829
C60	6202.34938	6202.362854	2.172327338
C61	6331.39197	6331.405314	2.107519671
C62	6459.45055	6459.457774	1.118289096
C63	6558.51896	6558.527534	1.307236157
C64	6659.56664	6659.582714	2.413600462
C65	6773.60957	6773.619204	1.422215588
C66	6872.67798	6872.689834	1.724732798
C67	6929.69944	6929.716804	2.505669008
C68	6986.7209	6986.735054	2.025776229
C69	7057.75801	7057.759284	0.18044443
C70	7156.82642	7156.836674	1.432692721
C73	7413.96397	7413.975164	1.509790601
C75	7614.08006	7614.091654	1.522643974
C76	7685.11717	7685.125564	1.092180241
C80	8111.37623	8111.395084	2.32433222
C139	14437.20285	14437.33742	9.321302369
Z34	3840.55795	3840.574224	4.237283575
Z44	4939.08848	4939.133244	9.063116261
Z46	5166.25185	5166.286294	6.667025557
Z50	5542.42651	5542.448244	3.921302895
Z51	5613.46362	5613.518074	9.700523029
Z52	5684.50073	5684.530244	5.191930571
Z54	5884.61682	5884.653474	6.228703455
Z55	5941.63828	5941.677064	6.527414038
Z56	6012.67539	6012.717814	7.055683264
Z57	6069.69685	6069.736294	6.49843544
Z60	6398.85553	6398.898774	6.75801054
Z61	6526.95049	6526.994704	6.77399548

Z62	6655.00907	6655.056584	7.139514405
Z63	6726.04618	6726.094644	7.20535242
Z64	6825.11459	6825.167444	7.743977397
Z65	6896.1517	6896.198724	6.818807817
Z67	7096.26779	7096.332684	9.144741298
Z68	7153.28925	7153.337344	6.723275327
Z70	7353.40534	7353.457904	7.148189266
Z71	7452.47375	7452.529504	7.481211607
Z72	7523.51086	7523.550024	5.205486355
Z73	7580.53232	7580.571194	5.12807432
Z74	7637.55378	7637.594964	5.392241326
Z76	7850.66512	7850.718914	6.852098809
Z77	7951.7128	7951.761074	6.070834591
Z78	8050.78121	8050.830904	6.172510695
Z79	8178.83979	8178.896914	6.984307626
Z80	8307.88238	8307.938994	6.814436042
Z81	8435.97734	8436.026484	5.825470024
Z82	8537.02502	8537.086384	7.187929399
Z84	8794.16257	8794.205504	4.882049061
Z85	8865.19968	8865.244864	5.096730446
Z86	8964.26809	8964.331344	7.056184898
Z87	9065.31577	9065.368424	5.808240381
Z88	9194.35836	9194.417044	6.382558829
Z89	9293.42677	9293.490554	6.863295392
Z90	9350.44823	9350.505184	6.090994969
Z92	9586.57555	9586.632414	5.931579303
Z93	9685.64396	9685.699304	5.713975586
Z94	9742.66542	9742.725684	6.185528346
Z96	9999.80297	9999.858144	5.517462023
Z97	10100.85065	10100.9142	6.291899101
Z98	10228.94561	10229.00332	5.642178121
Z101	10472.06751	10472.12658	5.641057324
Z102	10635.13084	10635.188	5.374972248
Z103	10748.2149	10748.27551	5.639404653
Z107	11161.44232	11161.50253	5.394780656
Z108	11262.49	11262.57937	7.935503883
Z110	11447.60642	11447.67109	5.649524516
Z111	11518.64353	11518.71914	6.564447709
Z113	11718.72323	11718.78545	5.309753623
Z114	11789.76034	11789.79952	3.323522446

Z115	11888.82875	11888.89158	5.285090268
Z117	12073.90879	12073.96222	4.425537252
Z118	12202.00375	12202.06159	4.74049462
Z119	12303.05143	12303.10353	4.235008967
Z120	12431.14639	12431.21867	5.814711762
Z121	12560.18898	12560.25666	5.388735252
Z122	12631.22609	12631.29203	5.220675543
Z124	12773.30031	12773.34531	3.52325022
Z126	12971.43713	12971.49284	4.295093333
Z127	13028.45859	13028.52529	5.119833069
Z128	13157.50118	13157.55934	4.420560738
Z129	13285.59614	13285.64702	3.829977413
Z131	13484.72821	13484.78553	4.250996553
Z132	13571.76024	13571.82133	4.501518746
Z133	13684.8443	13684.91114	4.884493507
Z134	13741.86576	13741.92519	4.32499736
Z136	14001.00121	14001.06873	4.822764609
Z139	14362.16497	14362.22847	4.421584995

Table S4. Electron capture dissociation (ECD) fragment assignment list for isotopically standard WT dimer, $[2M+19H]^{19+}$ charge state, processed and analyzed using DataAnalysis v4.2 and ProSight Lite.

Assigned Ion	Theoretical Neutral Mass (Da)	Observed Neutral Mass (Da)	Mass Error (ppm)
C4	509.23055	509.2304735	-0.150161611
C5	640.27104	640.2710335	-0.010100222
C6	768.366	768.3662635	0.342978633
C7	825.38746	825.3877135	0.307168611
C9	1025.50355	1025.504094	0.530015834
C11	1224.63562	1224.637334	1.399218749
C13	1481.77317	1481.775314	1.446600035
C17	1807.96856	1807.969234	0.372535859
C18	1879.00567	1879.006404	0.390383665
C20	2079.08537	2079.085264	-0.051208517
C21	2207.18033	2207.180564	0.10580609
C22	2308.22801	2308.231094	1.335887576
C23	2436.32297	2436.323974	0.411904798
C24	2564.38155	2564.385454	1.522212293
C27	2791.50853	2791.511274	0.982813805
C28	2920.55112	2920.554804	1.261245898
C31	3119.6468	3119.654534	2.478977146
C35	3605.92699	3605.931484	1.246151997
C36	3662.94845	3662.953834	1.469726695
C37	3762.01686	3762.024264	1.96796915
C38	3875.10092	3875.106584	1.461518871
C39	4038.16425	4038.168724	1.107813562
C44	4510.42879	4510.434154	1.189140405
C46	4767.56634	4767.573284	1.456410383
C48	4923.65621	4923.658914	0.549090555
C50	5159.78353	5159.788314	0.927080195
C53	5386.91051	5386.915494	0.925118973
C55	5587.0266	5587.026694	0.016741127
C56	5658.06371	5658.066494	0.491958603
C57	5787.1063	5787.109614	0.57257167
C60	6144.3439	6144.347384	0.566949568
C61	6273.38649	6273.386834	0.054760395
C63	6500.51348	6500.518364	0.751253441
C75	7556.07458	7556.082944	1.106862172

C92	9081.88456	9081.896594	1.325003973

Table S5. Electron capture dissociation (ECD) fragment assignment list for isotopically standard A53E dimer, $[2M+19H]^{19+}$ charge state, processed and analyzed using DataAnalysis v4.2 and ProSight Lite.

Assigned Ion	Theoretical Neutral Mass (Da)	Observed Neutral Mass (Da)	Mass Error (ppm)
C5	640.27104	640.2709835	-0.08819215
C6	768.366	768.3662435	0.316949371
C7	825.38746	825.3877235	0.319284134
C8	938.47152	938.4723335	0.866870334
C13	1481.77317	1481.778774	3.78164029
C21	2207.18033	2207.184454	1.868235714
C22	2308.22801	2308.230754	1.188588436
C23	2436.32297	2436.330334	3.022396132
C24	2564.38155	2564.387934	2.489307069
C27	2791.50853	2791.514924	2.290350558
C28	2920.55112	2920.556054	1.689247309
C31	3119.6468	3119.659504	4.072106214
C32	3247.74176	3247.746674	1.512907578
C37	3762.01686	3762.026114	2.459726648
C38	3875.10092	3875.109734	2.274400926
C39	4038.16425	4038.172974	2.16027199
C44	4510.42879	4510.433734	1.096022872
C50	5159.78353	5159.797454	2.698472337
C60	6202.34938	6202.351894	0.405255003
C61	6331.39197	6331.397804	0.921366604
